# Amyloid β1-42 (Aβ1-42) Induces the CDK2-Mediated Phosphorylation of Tau through the Activation of the mTORC1 Signaling Pathway While Promoting Neuronal Cell Death

**DOI:** 10.3389/fnmol.2017.00229

**Published:** 2017-07-24

**Authors:** Ki Hoon Lee, Sei-Jung Lee, Hyun Jik Lee, Gee Euhn Choi, Young Hyun Jung, Dah Ihm Kim, Amr Ahmed Gabr, Jung Min Ryu, Ho Jae Han

**Affiliations:** ^1^Department of Veterinary Physiology, College of Veterinary Medicine, Research Institute for Veterinary Science and BK21 PLUS Program for Creative Veterinary Science Research Center, Seoul National University Seoul, South Korea; ^2^Department of Pharmaceutical Engineering, Daegu Haany University Gyeongsan, South Korea; ^3^Department of Physiology, Faculty of Veterinary Medicine, Cairo University Giza, Egypt; ^4^Department of Veterinary Physiology, College of Veterinary Medicine, Chonnam National University Gwangju, South Korea

**Keywords:** Alzheimer’s disease, mammalian target of rapamycin, cyclin dependent kinase 2, tauopathy, neuronal cell death

## Abstract

Alzheimer’s disease (AD) is a neurodegenerative disorder, characterized by cognitive impairment and memory loss. Amyloid β1-42 (Aβ) and hyper-phosphorylation of microtubule-associated protein tau have been considered as major histological features in AD. However, the mechanism of how Aβ induces the hyper-phosphorylation of tau remains to be clarified. In the present study, we investigated the underlying cellular mechanisms of Aβ with regard to the cell cycle regulatory protein-mediated phosphorylation of tau in promoting neuronal cell death. The oligomer Aβ (5 μM) significantly increased the level of caspase 3 cleavage and has the ability to induce cytotoxicity in human neuroblastoma SK-N-MC cells. Aβ induced the degree of extracellular calcium influx via the L-type channel to facilitate the production of reactive oxygen species (ROS). Aβ signaling through ROS production is uniquely mediated by the activation of PI3K/Akt, which is in turn required for mammalian target of rapamycin complex 1 (mTORC1) phosphorylation. mTORC1 activated by Aβ further increased the phosphorylation of eukaryotic translation initiation factor 4E (eIF4E), a binding protein (4E-BP1) and p70S6K1 to stimulate the HIF1α synthesis responsible for the induction of cyclinD_1_/cyclin-dependent kinase 4 (CDK4) and cyclinE/CDK2, whereas it significantly attenuated the activation of autophagy. Aβ distinctively induced the CDK2-mediated phosphorylation of tau, which is responsible for microtubule destabilization in promoting neuronal apoptosis. In mouse hippocampal primary neurons, the apoptotic cell death induced by Aβ is highly susceptible to the mTORC1 signaling pathway. These results demonstrate that Aβ efficiently stimulates the mTORC1 signaling pathway to facilitate HIF1α synthesis and autophagy inhibition to promote the expression of cell cycle regulatory proteins, during which CDK2 uniquely stimulates tau phosphorylation for microtubule destabilization-mediated neuronal apoptosis.

## Introduction

Alzheimer’s disease (AD) is a common aging-associated disease causing cognitive impairment and memory loss. Two histopathological hallmarks are generally found in most AD patients: Amyloid β1-42 (Aβ) deposition and neurofibrillary tangles (NFTs; Spires-Jones and Hyman, 2014; Kim et al., 2017). Senile plaque is an extracellular accumulation of Aβ which is composed of soluble oligomer Aβ, while NFTs are intraneuronal aggregates of the highly phosphorylated microtubule-associated protein tau, also referred to as tauopathy (Spires-Jones and Hyman, 2014). In AD, however, tau is abnormally hyper-phosphorylated and detached from the microtubule, which leads to microtubule destabilization (Johnson and Stoothoff, 2004). The increased and detached soluble tau undergoes conformational changes and forms paired helical filaments which are believed to be toxic to cells (Wischik et al., 1995; Hyman et al., 2005). Previous research has suggested that tauopathy is induced by specific phosphorylation sites in tau, such as Thr 212 and Ser 396 (Alonso et al., 2010). Thus, many researchers have recently focused on elucidating how Aβ induces the hyper-phosphorylation of tau, which is a critical therapeutic target of tauopathy in AD patients.

A nutrient-sensing serine/threonine protein kinase, the mammalian target of rapamycin (mTOR), was shown to be hyper-phosphorylated in AD patients. In addition, previous studies have discovered that mTOR is a critical candidate molecule; it phosphorylates tau protein (Sun et al., 2014; Tramutola et al., 2015), and the inhibition of mTOR can ameliorate AD symptoms (Wang et al., 2014). On the other hand, healthy neurons are not believed to proceed to the G1/S phase and do not replicate DNA (Copani et al., 2007). However, cell cycle regulatory proteins are increased in AD patients which is associated with cell cycle re-entry process (Moh et al., 2011). And, the dysregulation of cell cycle regulator protein could be involved in the microtubule-mediated mechanisms associated with neuroplasticity (Arendt and Brückner, 2007). Although Aβ is known to increase cell cycle regulatory proteins through reactive oxygen species (ROS)-mediated signaling, the mechanism acting between Aβ and the accumulation of cell cycle regulatory proteins is poorly understood (Folch et al., 2012). Hence, discovering the functional mechanism by which Aβ regulates cell cycle re-entry by exploiting the mTOR signaling pathway serves as a novel target for the treatment and/or prevention of AD pathogenesis. Autophagy regulation is also another major role of mTOR. Autophagy is a conserved physiological pathway consisting of several steps, including initiation, autophagosome formation, fusion with lysosomes, and degradation (Klionsky and Emr, 2000; He and Klionsky, 2009). Recent reports have shown that hyper-activated mTOR in AD patients accelerates apoptosis by inhibiting autophagy (Son et al., 2012; Liang and Jia, 2014). Given that the increased expression of cell cycle regulatory proteins is toxic to neuronal cells and that toxic molecules are degraded by autophagy for cell survival, we investigated the roles and interplay of mTOR, cell cycle re-entry, and autophagy in the tauopathy of AD.

In the present study, we used the neuroblastoma SK-N-MC cell line and mouse hippocampal primary neurons to study the role of Aβ in promoting neuronal cell death. SK-N-MC neuronal cell line has been widely used for studying the pathogenesis and molecular mechanism of neurodegenerative diseases including AD due to its advantages, such as high stability and homogeneity (Chan et al., 2007; Shaykhalishahi et al., 2010; Kuo and Chou, 2014; Lee et al., 2016). In addition, the hippocampus, a major component of the brains of vertebrates, is known as the first area of the brain affected by the pathogenic mechanisms occurring in AD. In the present study, therefore, we investigated the underlying cellular mechanisms of Aβ with regard to the cell cycle regulator-mediated AD pathogenesis with SK-N-MC cells and mouse hippocampal primary neurons.

## Materials and Methods

### Materials

The neuroblastoma SK-N-MC cell lines were obtained by Korean Cell Line Bank (Seoul, South Korea). Fetal bovine serum (FBS) was purchased from Hyclone (Logan, MD, USA). The antibodies of p-Akt (Thr 308, sc-16646-R), p-Akt (Ser 473, sc-7985-R), Akt (sc-8312), eukaryotic translation initiation factor 4E (eIF4E; sc-9976), cyclin D_1_ (sc-753), cyclin E (sc-481), cyclin-dependent kinase 4 (CDK4; sc-749), CDK2 (sc-748), tau (sc-390476), p-tau (Thr 212, sc-135643), p-tau (Ser 396, sc-101815), rapamycin-insensitive companion of mTOR (RICTOR; sc-271081), α-tubulin (sc-32293) and β-actin (sc-47778) were purchased from Santa Cruz Biotechnology (Dallas, TX, USA). Horseradish peroxidase (HRP)-conjugated IgG was obtained from Jackson Immunoresearch (West Groove, PA, USA). The antibodies of cleaved caspase 3(#9661), mammalian target of rapamycin (mTOR; #2983), p-mTOR (Ser 2448, #5536), p-mTOR (Ser 2481, #2974), p-regulatory-associated protein of mTOR (RAPTOR; Ser 792, #2083), p70 S6 kinase 1 (S6K1, #2708), p-p70S6K1 (Thr 389, #9234), eIF4E binding protein (4EBP1; #9644), p-4EBP1 (Thr 37/46, #9459) and p35/25 (#2680) were acquired from Cell Signaling Technology (Beverly, MA, USA). The antibodies of hypoxia inducible factor (HIF1α; NB100-105), p70S6K1 (NB600-1049), LC3 (NB100-2220) and p62 (NBP1-48320) were obtained from Novus Biologicals (Littleton, CO, USA) and the HRP-conjugated goat anti-rabbit IgG was purchased from Santa Cruz Biotechnology. NeuN (ab77315), RAPTOR (ab26264) antibodies were purchased from Abcam (Cambridge, England). Small interfering RNA (siRNA) for *HIF1A* was purchased from GenePharma (Sanghai, China). *CDK2*, *CDK4* and non-targeting (NT) siRNAs were purchased from Dharmacon (Lafayette, CO, USA). EGTA, BAPTA-AM, ionomycin, nifedipine, *N-acetyl-L-cysteine* (NAC), PF4708671, cycloheximide, LY294002, rapamycin, trehalose, flavopiridol, paclitaxel were purchased from Sigma Aldrich (St. Louis, MO, USA). Akt inhibitor was purchased from Calbiochem (La Jolla, CA, USA). All chemicals used in this study were of the highest quality commercially available forms.

### Cell Culture

The SK-N-MC cells were cultured with Dulbecco Modified Eagle Medium (DMEM, Thermo Fisher, Waltham, MA, USA). Cells were grown in 10% FBS with a 1% antibiotics mixture. Cells were plated on 35, 60, or 100 mm diameter culture dishes, or in 6- or 12-well plates in an incubator kept at 37°C with 5% CO_2_. The medium was changed to serum-free medium. After 24 h of incubation in serum-free medium, cells were washed twice with phosphate buffered solution (PBS), and placed in medium that included supplements.

### Primary Culture of Hippocampal Neurons from Mice

Hippocampal primary neurons were isolated from prenatal ICR mice (18–19 days) to confirm the effect of Aβ in neurons. Isolation was performed as described (Seibenhener and Wooten, 2012). Hippocampus was isolated from the prenatal mice (18–19 days), and gently minced by using sterile scalpel. Minced hippocampus was treated with trypsin (0.025%) and dissociated by trituration. 2.5 × 10^6^ cells were plated at poly-D-lysine-coated 35 mm dish in neurobasal plating media (neurobasal Media containing B27 Supplement [1 ml/50 ml], 0.5 mM glutamine, 25 μM glutamate, 1% antibiotics, 1 mM HEPES, 10% heat inactivated donor horse serum) and placed in an incubator kept at 37°C with 5% CO_2._ After 24 h, growth media is changed to neurobasal feeding media (Neurobasal media containing B27 supplement [1 ml/50 ml], 0.5 mM glutamine, 1% antibiotics, 1 mM HEPES. To avoid glial cell contamination, cytosine arabinoside (AraC) was treated early time points in the culture. NeuN antibody was used for neuronal nucleus staining. The protocol for mouse hippocampal neuron primary culture was approved by the Institutional Animal Care and Use Committee of Seoul National University (SNU-151116-1). All procedures involving mice were performed following the National Institutes of Health Guidelines for Humane Treatment of Animals.

### Preparation of Aβ

The β-Amyloid [1-42] (Human) peptide was purchased from Invitrogen Corporation (Camarillo, CA, USA). Peptide was dissolved at a concentration of 1 mg/ml in 100% HFIP (Sigma Aldrich), and incubated at room temperature for 1 h with occasional vortexing. The sonicated for 10 min in a water bath sonciator. After sonication, it was freeze-dried for 3 h. When the film is formed, it dissolved in DMSO. Scrambled β-Amyloid [1-42] (scrambled Aβ) was purchased from ANASPEC (San Jose, CA, USA). Protein sequence of scrambled Aβ is as follows. AIAEGDSHVLKEGAYMEIFDVQGHVFGGKIFRVV DLGSHNVA.

### Real Time Polymerase Chain Reaction (Real Time-PCR)

The total RNA of cells was extracted using MiniBEST Universal RNA Extraction Kit (TaKaRa, Otsu, Shinga, Japan). Reverse transcription (RT) was carried out using a Maxime™ RT-PCR PreMix kit (Intron Biotechnology, Seongnam, Korea) with the oligo(dT18) primers. RT was performed at 45°C for 60 min to cDNA synthesis and 95°C for 5 min RTase inactivation step. RT products were amplified using QuantiSpeed SYBR Kits (Life technologies, Gaithersburg, MD, USA). Real-time quantification of RNA targets was performed in a Rotor-Gene 6000 real-time thermal cycling system (Corbett Research, NSW, Australia). The primers used are described in the Supplementary Table S1. The reaction mixture (10 μl) contained 50 ng of total RNA, 0.5 mM of each primer, and appropriate amounts of enzymes and fluorescent dyes as recommended by the supplier. The Real-time PCR were performed as follows: 15 min at 95°C for DNA polymerase activation; 15 s at 95°C for denaturing; and 40 cycles of 15 s at 94°C, 30 s at 54°C and 30 s at 72°C. Data was collected during the extension step (30 s at 72°C) and analysis was performed using the software provided. Following real-time PCR, melting curve analysis was performed to verify the specificity and identity of the PCR products.

### Western Blot Analysis

Harvested cells were washed once with cold PBS prior to incubation in lysis buffer (1 mM EDTA, 1 mM EGTA, 20 mM Tris (pH 7.5), 1% Triton X-100, 1 mg/ml aprotinin, and 1 mM phenylmethylsulfonylfluoride (PMSF)) for 30 min on ice. The lysates were then cleared by centrifugation (16,000 *g* at 4°C for 30 min). The BCA assay with lysates was performed to determine protein concentration. Samples containing 10 μg of protein were prepared for 10% sodium dodecyl sulfate polyacryl-amide gel electrophoresis (SDS-PAGE) and then transferred to a polyvinylidene fluoride (PVDF) membrane. Protein-containing membranes were washed Tris-buffered saline containing 0.1% Tween-20 (TBST) solution (10 mM Tris-HCl (pH 7.6), 150 mM NaCl and 0.1% Tween-20) blocked with 5% bovine serum albumin (BSA) for 30 min. Blocked membranes were washed with TBST for three times every 10 min, and incubated with primary antibody overnight at 4°C. The membranes were then washed and incubated with HRP-conjugated secondary antibody at room temperature for 2 h. The western blotting bands were visualized by using chemiluminescence (Bio-Rad, Hercules, CA, USA). Densitometric analysis was carried out by using ImageJ software (developed by Wayne Rasband, National Institutes of Health, Bethesda, MD, USA^1^).

### Transfection of Small Interfering RNA

Prior to Aβ treatment, siRNAs specific for *HIF1A*, *CDK2*, *CDK4*, or NT were transfected to cell for 24 h with Turbofect™ (Thermo Fisher) according to the manufacturer’s instructions. The concentration of each transfected siRNA was 25 nM. The NT siRNA was used as the negative control. The siRNA sequences are described in Supplementary Table S2.

### Trypan Blue Exclusion Test for Measuring Cell Viability

Cells were incubated with a 0.05% Trypsin and 0.5 mM EDTA solution. Detached cells were treated with soybean trypsin inhibitor (0.05 mg/ml) to quench trypsin. To exclude dead cells, 0.4% trypan blue was added to the cell suspension. Subsequently, unstained and stained cells were counted by using a Petroff-Hausser counting chamber (Hausser Scientific, Horsham, PA, USA). The equation used to determine cell viability was Cell viability = [{1 − (number of trypan blue-stained cells/number of total cells)}] × 100.

### 3-(4,5-Dimethylthiazol-2-yl)-2,5-Diphenyltetrazolium Bromide (MTT) Assay

The cells were cultured in a 12-well plate. Prior to harvesting, each well was treated with 100 pg/ml of MTT reagent (Sigma Aldrich) for 4 h. Harvested cells were washed with PBS and incubated with 150 μl of DMSO (Sigma Aldrich) for 20 min. 1 × 10^5^ cells were collected, and resuspended in 150 μl of PBS. Absorbance at 540 nm was measured by using a micro plate reader (Bio-Rad).

### Immunofluorescence Staining

Cells were fixed with 4% paraformaldehyde (Sigma Aldrich) for 10 min, followed by permeabilization with 0.1% Triton X-100 (Sigma Aldrich) in PBS followed by washing with PBS. Cells were incubated with 1% normal goat serum to block non-specific binding of antibody, and then incubated with 1:100 dilution of primary antibody for overnight in 4°C. Next, the cells were incubated for 2 h at room temperature with Alexa Fluor^®^-conjugated secondary antibodies (1:100 dilution) and propidium iodide (PI, Invitrogen, Carlsbad, CA, USA) in PBS. Images were obtained by using a FluoView™ 300 fluorescence microscope (Olympus, Tokyo, Japan). Three independent experiments were performed and 60 cells (20 cells per experiment) were used for statistical analysis.

### Measurement of [Ca^2+^]_i_

The changes in [Ca^2+^]_i_ were observed using Fluo 3-AM dissolved in DMSO. The cells were washed once with a PBS, incubated in PBS containing 3 μM of Fluo 3-AM with 5% CO_2_ at 37°C for 40 min, and washed once with the PBS and scanned every second using confocal microscopy (FluoView™ 300, Olympus, Tokyo, Japan). The fluorescence was excited at 488 nm and the emitted light was read at 515 nm. In order to verify the assay, ionomycin was applied to the cells as a positive control. All of the analysis of [Ca^2+^]_i_ were processed at a single cell level are expressed as the relative fluorescence intensity (RFI).

### Measurements of Intracellular ROS Levels

The cells were plated on 12-well dish and confocal dishes. The cells were then incubated in the dark with 10 μM CM-H_2_DCFDA for 1 h at 37°C. The cells were visualized by using laser confocal microscopy (FluoView™ 300, Olympus, Tokyo, Japan) with an excitation wavelength of 448 nm and an emission wavelength of 515 nm. To quantify the ROS production, cells were incubated with 10 μM of CM-H_2_DCFDA, and washed twice with PBS. The 1 × 10^5^ cells were loaded into a 96-well black plate and assessed by using a luminometer (Victor3, Perkin-Elmer, Waltham, MA, USA).

### Co-Immunoprecipitation

Cells were lysed with the co-immunoprecipitation buffer (1% Triton X-100 in 50 mM Tris-HCl [pH 7.4] containing 150 mM NaCl, 5 mM EDTA, 2 mM NA_3_VO_4_, 2.5 mM Na_4_PO_7_, 100 mM NaF, 200 nM microcystin lysin-arginine, and protease inhibitor). Cell lysates (300 μg) were incubated with 10 μg of anti-tau antibody. The samples were incubated for 4 h, and Protein A/G PLUS-agarose immunoprecipitation reagent was added. The samples were incubated for an additional 12 h. The beads were washed three times with the co-immunoprecipitation buffer. Beads were added to sample buffer, and eluted by boiling. Samples were analyzed by western blotting.

### AnnexinV/PI Double Staining

Apoptosis and necrosis of cells were measured with an Annexin V and PI staining kit (BD Biosciences, Franklin Lakes, NJ, USA) according to the manufacturer’s instructions. Briefly, the cells were detached with 0.05% trypsin/EDTA and 1 × 10^5^ cells were suspended with Annexin V binding buffer. The cells were stained with Annexin V and PI and incubated for 20 min at room temperature in the dark. The sample was read by flow cytometry and analyzed using CXP software (Beckman Coulter, Brea, CA, USA). Samples were gated to exclude debris (forward light scatter [FSC] area vs. side scatter area), and then any cell doublets were excluded using FSC area vs. FSC width analysis.

### Statistical Analysis

Results are expressed as mean value ± standard error of mean (SE). All experiments were analyzed by analysis of variance (ANOVA), and some experiments which needed to compare with two groups were examined by comparing the treatment means to the control using a Student’s test. A result with a *p* value of < 0.05 was considered statistically significant.

## Results

### Effect of Aβ on Neuronal Cell Death

**Figure 1:**
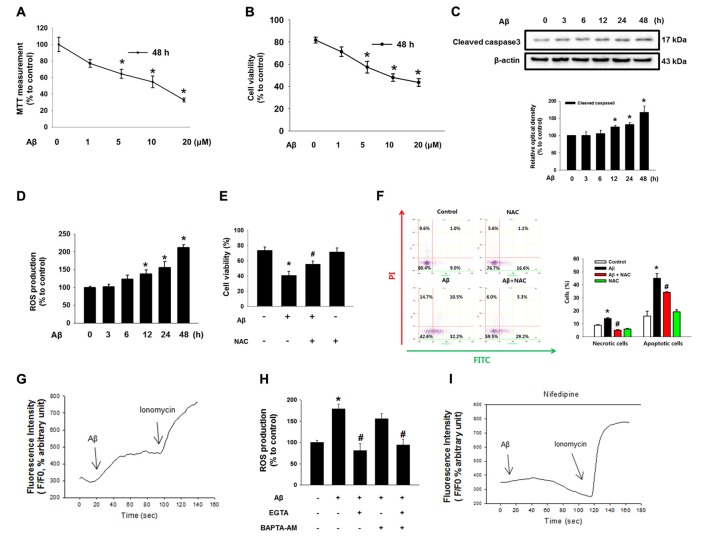
Effect of amyloid β (Aβ) on neuronal cell death. **(A)** SK-N-MC cells were exposed to various concentration of Aβ (0–20 μM) for 48 h. Cytotoxicity was measured by MTT assay at an absorbance of 545 nm using a microplate reader. Data are presented as a mean ± SE. *n* = 6. **(B)** Cell viability was measured using a counting chamber by trypan blue exclusion assay. Data are presented as a mean ± SE. *n* = *6*. **(C)** Cells were exposed to Aβ (5 μM) for 0–48 h. Cleaved caspase-3 was detected by western blot. *n* = 4. **(D)** Reactive oxygen species (ROS) measurement by CM-H_2_DCFDA was conducted by using luminometer. Data are presented as a mean ± SE. *n* = 6. **(E)** Cells were treated with Aβ (5 μM) and N-acetyl-L-cysteine (NAC; 1 mM) for 24 h. Cell viability was measured by trypan blue exclusion assay. Data represent the mean ± SE. *n* = 6. **(F)** Necrotic and apoptotic cells were counted by using annexin V/PI analysis with flow cytometry. Data are presented as a mean ± SE. *n* = 4. **(G,I)** SK-N-MC cells were loaded with 2 μM of Fluo-3 AM for 40 min, subsequently pretreated with Nifedipine (10 μM) for 30 min prior to Aβ treatment for 24 h. **(H)** Cells were pretreated with EGTA (1 mM) and BAPTA-AM (10 μM) for 30 min prior to Aβ treatment. ROS measurement was performed by using luminometer. Data are presented as a mean ± SE. *n* = 6. Each blot result shown is representative image. Quantitative blot data are presented as a mean ± SE. *n* = 4. *p < 0.05 vs. control, ^#^*p* < 0.05 vs. Aβ treatment.

Aβ significantly induced the cytotoxicity of SK-N-MC cells from 1 μM to 20 μM for 48 h (Figure 1A). For instance, cell viability decreased by 36% at 5 μM of Aβ compared to a control sample. The results of a trypan blue exclusion assay also revealed that cell viability decreased by 34% when the cells were treated with 5 μM of Aβ (Figure 1B). In addition, we found that Aβ has the ability to induce caspase 3 cleavage, suggesting that neuronal cell death as induced by Aβ is related to apoptotic cell death (Figure 1C). It was also found that Aβ increased ROS production by as much as 87% in 3 h, with the rate strongly maintained for up to 48 h (Figure 1D). The stimulatory effect of Aβ on ROS production was further visualized with a florescent dye, CM-H_2_DCFDA (Supplementary Figure S1A). Importantly, the cell viability decreased by the Aβ treatment was significantly recovered by a treatment with the ROS scavenger NAC (Figure 1E). Consistent with this, we found through flow cytometric analyses that Aβ significantly induced the necrotic cell death (a 1.3 ± 0.8-fold increase compared to a control) as well as apoptosis (a 3.0 ± 0.2-fold increase compared to a control; Figure 1F). In addition, we performed CM-H_2_DCFDA staining to confirm the effect of NAC in ROS production. We observed that ROS level of Aβ-treated SK-N-MC was increased to 143%. And, those of NAC-pretreated SK-N-MC with either vehicle or Aβ were decreased to 85% and 72%, respectively. (Supplementary Figure S1B). Aβ has greater stimulatory potency on apoptotic cell death than necrotic cell death, confirming that Aβ is essential for triggering apoptotic cell death. However, this increase in apoptotic cell death was significantly blocked by a treatment with NAC. Next, we determine whether ROS production induced by Aβ causes alterations in the Ca^2+^ influx [Ca^2+^]_i_ level. As shown in Figure 1G, Aβ caused an increased level of [Ca^2+^]_i_, which was enhanced by the Ca^2+^ ionophore ionomycin as a positive control. However, ROS production as induced by Aβ was significantly blocked by a pretreatment with the extracellular calcium chelator EGTA but not with the intracellular calcium chelator BAPTA-AM in SK-N-MC cells, indicating that Aβ-induced ROS is dependent on extracellular [Ca^2+^]_i_ (Figure 1H). Interestingly, the [Ca^2+^]_i_ induced by Aβ was blocked by a treatment with an L-type calcium channel blocker, Nifedipine (Figure 1I and Supplementary Figure S2). These results suggest that [Ca^2+^]_i_ is highly dependent on extracellular calcium influx.

### The Role of Aβ in the Activation of mTORC1

**Figure 2:**
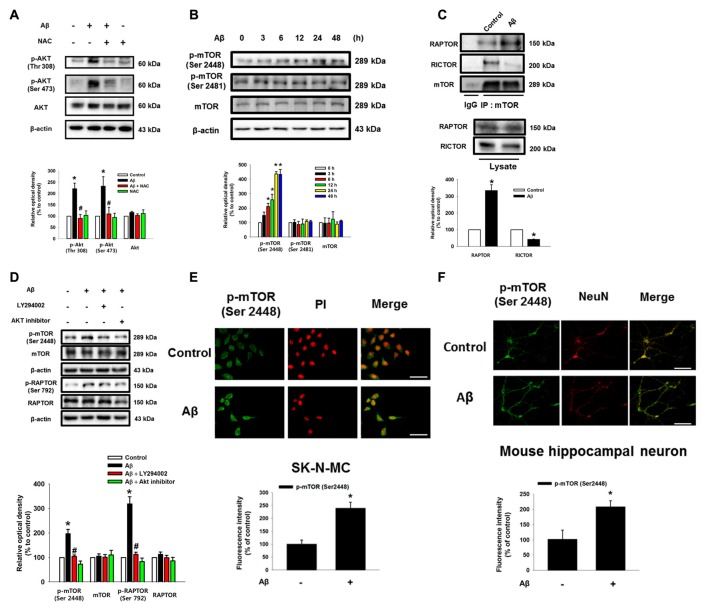
Role of Aβ in the activation of Akt and mammalian target of rapamycin complex 1 (mTORC1). **(A)** SK-N-MC were incubated with Aβ (5 μM) and NAC (1 mM) for 24 h. Phosphorylated Akt (Thr 308 or Ser 473), Akt and β-actin were detected by western blot. *n* = 6. **(B)** Cells were incubated with Aβ for 0–48 h. Phosphorylated mTOR (Ser 2448 and Ser 2481), mTOR and β-actin were detected by western blot. *n* = 3. **(C)** Cells were treated with Aβ for 24 h. Protein samples were immunoprecipitated by using mTOR antibody-conjugated protein A/G agarose beads. Immunoprecipitation assay was described in “Materials and Methods” section. Raptor, Rictor and mTOR were detected by western blot. *n* = 3. **(D)** Cells were pretreated with LY294002 (20 μM) or Akt inhibitor (20 μM) for 30 min prior to Aβ treatment for 24 h. Cells were blotted with p-mTOR (Ser 2448), mTOR, p-Raptor (Ser 792), Raptor and β-actin specific antibodies. *n* = 4. Each western blot image was presented as representative image. Quantitative blot data are presented as a mean ± SE. *n* = 3. *p < 0.05 vs. control, ^#^*p* < 0.05 vs. Aβ treatment. **(E,F)** SK-N-MC cells and mouse hippocampal neurons were immunostatined with p-mTOR (Ser 2448) specific antibodies, and visualized by confocal microscopy. Scale bars, 100 μm (magnification, ×400). Data are presented as a mean ± SE. *n* = 3.

**Figure 3:**
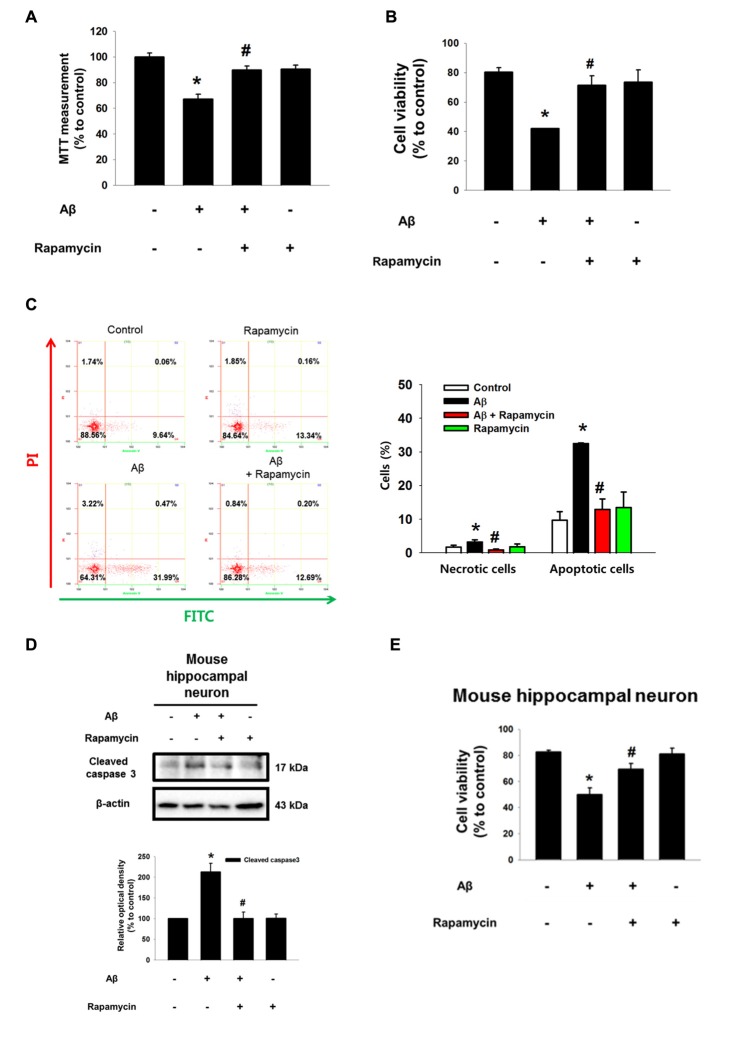
Role of mTORC1 in the neuronal cell death induced by Aβ. **(A)** SK-N-MC cells were pretreated to rapamycin 10 nM for 30 min prior to Aβ (5 μM) treatment for 24 h. Cytotoxicity was measured by MTT assay at an absorbance of 545 nm using a microplate reader. *n* = 6. **(B)** Cell viability was measured by trypan blue exclusion assay. *n* = 6. **(C)** Necrotic and apoptotic cells were counted by using annexin V/PI analysis with flow cytometry. *n* = 4. **(D)** Mouse hippocampal neurons were pretreated with rapamycin (10 nM) for 30 min prior to Aβ (5 μM) for 24 h. Samples were blotted with cleaved caspase-3 and β-actin specific antibodies. *n* = 6. **(E)** Cell viability of mouse hippocampal neuron was counted by trypan exclusion assay. Data are presented as a mean ± SE. *n* = 6. Each western blot image was presented as representative image. Quantitative blot data are presented as a mean ± SE. *p < 0.05 vs. control, ^#^*p* < 0.05 vs. Aβ treatment.

As shown in the Figure 2A and Supplementary Figure S3, Aβ significantly induced the phosphorylation of Akt at Thr 308 and Ser 473, which was blocked by NAC pretreatment in SK-N-MC and mouse hippocampal neuron. Aβ also significantly induced the phosphorylation of mTOR at Ser 2448, but not at Ser 2481 (Figure 2B). mTOR was more likely to bind RAPTOR, while decreasing the binding of RICTOR when the cells were treated with Aβ for 24 h, suggesting that Aβ uniquely regulates the phosphorylation of mTOR Complex 1 (mTORC1) rather than mTORC2 (Figure 2C). Importantly, the inhibition of PI3K with LY294002 and Akt with an Akt inhibitor significantly blocked the phosphorylation of mTORC1 and RAPTOR, suggesting that Aβ triggers mTORC1 activation through the PI3K/Akt pathway (Figure 2D). The mTOR phosphorylation at Ser 2448 residue in Aβ-treated SK-N-MC cells and hippocampal primary neurons were increased to 238% and 208%, respectively (Figures 2E,F). Given that Aβ specifically induces mTOR phosphorylation, we further assessed whether mTOR activation is involved in the regulation of neuronal apoptosis. The cytotoxicity (Figure 3A) and reduced number of cells (Figure 3B) induced by Aβ were significantly inhibited by a treatment with the mTOR inhibitor, rapamycin. The result in flow cytometric analyses also revealed that the rapamycin significantly blocked the apoptosis induced by Aβ (Figure 3C). Moreover, the levels of caspase-3 cleavage (Figure 3D) and cytotoxicity (Figure 3E) induced by Aβ in primary neurons were significantly blocked by rapamycin. These results indicate that Aβ stimulates the PI3K/Akt pathway required for mTORC1 activation to stimulate neuronal apoptosis.

### Aβ Facilitates HIF1α Synthesis and Autophagy Inhibition via mTOR Activation

**Figure 4:**
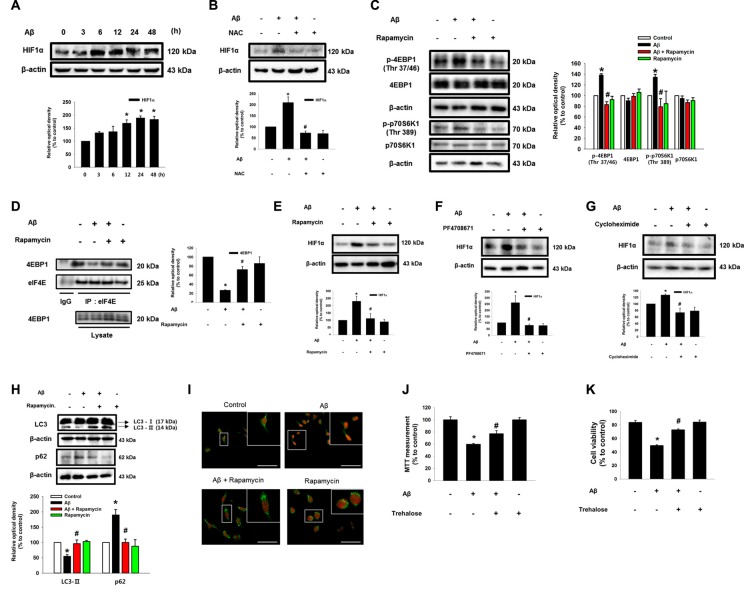
Aβ facilitates HIF1α synthesis and autophagy inhibition via mTOR activation. **(A)** SK-N-MC cells were exposed to Aβ (5 μM) for 0–48 h. HIF1α and β-actin expression was analyzed by western blot. *n* = 3. **(B)** Cells were pretreated with NAC (1 mM) for 30 min prior to Aβ treatment for 24 h. HIF1α and β-actin expression were analyzed by western blot. *n* = 3. **(C,E)** Cells were incubated with rapamycin (10 nM) for 30 min prior to Aβ treatment for 24 h. Phosphorylation of 4EBP1 (Thr 37/46) and 4EBP1, phosphorylation of p70S6K1 (Thr 389), HIF1α and β-actin were analyzed by western blot. *n* = 6. **(D)** Protein samples were immunoprecipitated by eukaryotic translation initiation factor 4E (eIF4E) antibody-conjugated protein A/G agarose beads. Samples were blotted with 4EBP1 and eIF4E-specific antibodies. *n* = 3. **(F)** Cells were exposed to PF4708671 (10 μM) for 30 min prior to Aβ treatment for 24 h. HIF1α and β-actin expression was detected by western blot. *n* = 6. **(G)** Cells were exposed to cycloheximide (4 μM) for 30 min prior to Aβ treatment for 24 h. HIF1α and β-actin expressions were detected by western blot. *n* = 6. **(H)** Cells were pretreated with rapamycin (10 nM) for 30 min, incubated with Aβ for 24 h and analyzed by western blotting with LC3, p62 and β-actin specific antibodies. *n* = 3–6. **(I)** LC3 puncta was visualized by confocal microscopy. Presented results are merged images. Green and red fluorescents indicate LC3 and PI respectively. Scale bars, 50 μm (magnification × 600). **(J)** Cells were pretreated with trehalose (10 μM) for 30 min prior to Aβ treatment for 24 h. Cytotoxicity was measured by MTT assay at an absorbance of 545 nm using a microplate reader. Data present the mean ± SE. *n* = 6. **(K)** Cell viability was measured by trypan blue exclusion assay. Data are presented as a mean ± SE. *n* = 6. Each blot image was presented as representative image. *p < 0.05 vs. control, ^#^*p* < 0.05 vs. Aβ treatment.

Despite the fact that Aβ is a strong regulator of ROS production, it was previously reported that Aβ reduces HIF1α expression via a ROS-independent mechanism in astrocytes (Schubert et al., 2009). However, our results showed that the protein level of HIF1α is increased by Aβ for 48 h in a time-dependent manner (Figure 4A). The increased HIF1α level was significantly inhibited by the ROS scavenger NAC (Figure 4B). To determine how ROS induced by Aβ regulates the level of the HIF1α protein, we focused on 4EBP1 and S6K1, which are involved in the mRNA translation of HIF1α. As shown in Figure 4C, Aβ induced the phosphorylation of 4EBP1 and p70S6K1, which was blocked by rapamycin, suggesting that mTOR activation is mediated by the activation of 4EBP1 and p70S6K1 in Aβ-treated SK-N-MC cells. In addition, Aβ-mediated 4EBP1 phosphorylation was closely related to a decreased level of 4EBP1 interaction with its binding protein eIF4E, in which a rapamycin treatment significantly recovered this interaction to the control level (Figure 4D). Importantly, the increased protein level of HIF1α induced by Aβ was markedly inhibited by a treatment with rapamycin (Figure 4E), the p70S6K1 inhibitor PF4708671 (Figure 4F), or the translation inhibitor cycloheximide (Figure 4G). These results indicate the novel role of Aβ in the promotion of HIF1α synthesis though the mTORC1 pathway, which is required for the phosphorylation of 4EBP1 and p70S6K1. Another major role of mTOR is known to be the inhibition of autophagy. As shown in Figure 4H, we found that Aβ reduces the expression of LC3-II and the degradation of p62, and that the inhibition of autophagy induced by Aβ significantly blocked by a treatment with rapamycin. We confirmed using confocal microscopy that Aβ inhibits the formation of autophagic vesicles via mTOR (Figure 4I). Interestingly, the cytotoxicity induced by Aβ was recovered by a treatment with the autophagy inducer trehalose (Figures 4J,K). Together, these results suggest that Aβ has the ability to induce two pathogenic pathways related to HIF1α synthesis and autophagy inhibition through the regulation of mTORC1 activation to promote neuronal apoptosis. Moreover, we pretreated inhibitors used in this study to demonstrate the sequential activation of Aβ-induced ROS/Akt/mTOR/HIF1α pathway. As shown in the Supplementary Figure S4, mTOR phosphorylation (Ser 2448) and HIF1α expression were suppressed by NAC or Akt inhibitor or rapamycin. However, Akt phosphorylations at Thr 308 and Ser 473 residues were inhibited by NAC or Akt inhibitor, but not rapamycin. These findings indicate the ROS-induced Akt is an upstream regulator of Akt/mTOR/HIF1α pathway.

### Aβ Induces the Expression of Cell Cycle Regulatory Proteins

**Figure 5:**
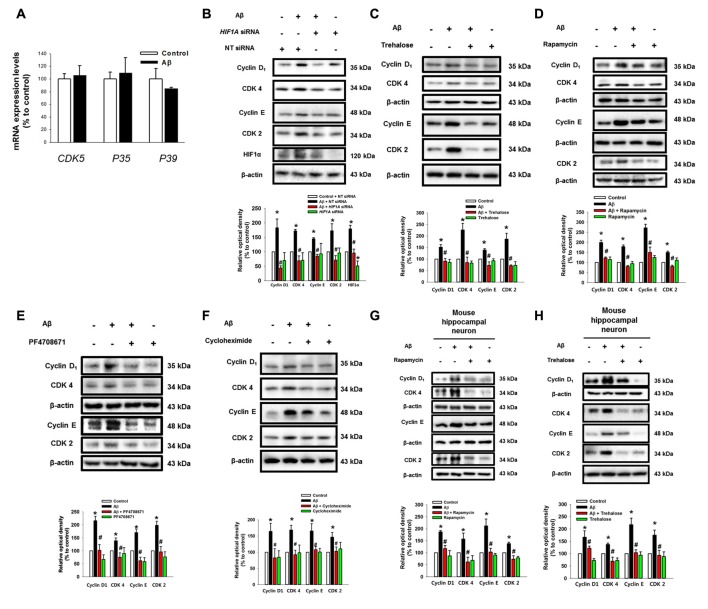
Aβ induces the expression of cell cycle regulatory proteins. **(A)** SK-N-MC cells were exposed to Aβ (5 μM) for 24 h. The mRNA expression levels of *CDK5*, *P35* and *P39* were analyzed by real-time PCR. The mRNA expression level was normalized by β-actin mRNA expression level. Data represent the mean ± SE. *n* = 4. **(B)** *hif1α* specific- and non-targeting (NT) siRNA were transfected to the cells for 24 h prior to Aβ treatment. Cyclin D_1_, CDK4, cyclin E, CDK2, HIF1α and β-actin was detected by western blot. *n* = 3. **(C–F)** Cells were pretreated with trehalose (10 μM), rapamycin (10 nM), PF4708671 (10 μM) and cycloheximide (4 μM) for 30 min prior to Aβ treatment for 24 h. Cyclin D_1_, CDK4, cyclin E, CDK2 and β-actin were detected by western blot. *n* = 3–6. **(G)** Mouse hippocampal neurons were transfected with *hif1α* specific- and NT siRNAs for 24 h prior to Aβ treatment for 24 h. Samples were blotted with Cyclin D_1_, CDK4, cyclin E, CDK2 and β-actin specific antibodies. *n* = 3–6. **(H)** Mouse hippocampal neurons were pretreated with trehalose (10 μM) for 30 min and incubated with Aβ for 24 h. cyclin D_1_, CDK4, cyclin E, CDK2, HIF1α and β-actin were analyzed by western blot. *n* = 3–6. Data are presented as a mean ± SE. *p < 0.05 vs. control, ^#^*p* < 0.05 vs. Aβ treatment.

To determine how two pathogenic pathways triggered by Aβ influence the neuronal cell death process, we subsequently investigated the role of Aβ at the level of cell cycle regulatory proteins including cyclin 2, cyclin 4, CDK2, CDK4 and CDK5, which are known as critical regulators of neuronal cell death in AD patients (Frade and Ovejero-Benito, 2015; Liu et al., 2016). Interestingly, Aβ did not have an effect on the mRNA expression of CDK5 or its regulators, p35 and p39, which are well-known candidate molecules responsible for the hyper-phosphorylation of tau protein in AD patients (Figure 5A). Moreover, our result showed that Aβ did not affect p35/p25 expression in SK-N-MCs (Supplementary Figure S5). Instead, we found that Aβ significantly induced the expression of cyclin D_1_/CDK4 and cyclin E/CDK2 (Figures 5B,C). However, the level of cell cycle regulatory proteins was inhibited by the knockdown of *HIF1A* (Figure 5B) and by a treatment with the trehalose (Figure 5C), respectively. In addition, the expression of cell cycle regulatory proteins induced by Aβ is markedly inhibited by a treatment with the rapamycin (Figure 5D), the p70S6K1 inhibitor PF4708671 (Figure 5E), and the translation inhibitor cycloheximide (Figure 5F). Importantly, our results with Aβ-treated primary hippocampal neurons revealed that the increase in the level of cell cycle regulatory proteins was blocked by a treatment with *HIF1A* siRNA (Figure 5G) and trehalose (Figure 5H). These results indicate that Aβ-induced mTOR signaling pathway stimulates the expression of cell cycle regulatory proteins.

### CDK2 Plays a Critical Role in Tau Phosphorylation and Microtubule Destabilization

**Figure 6:**
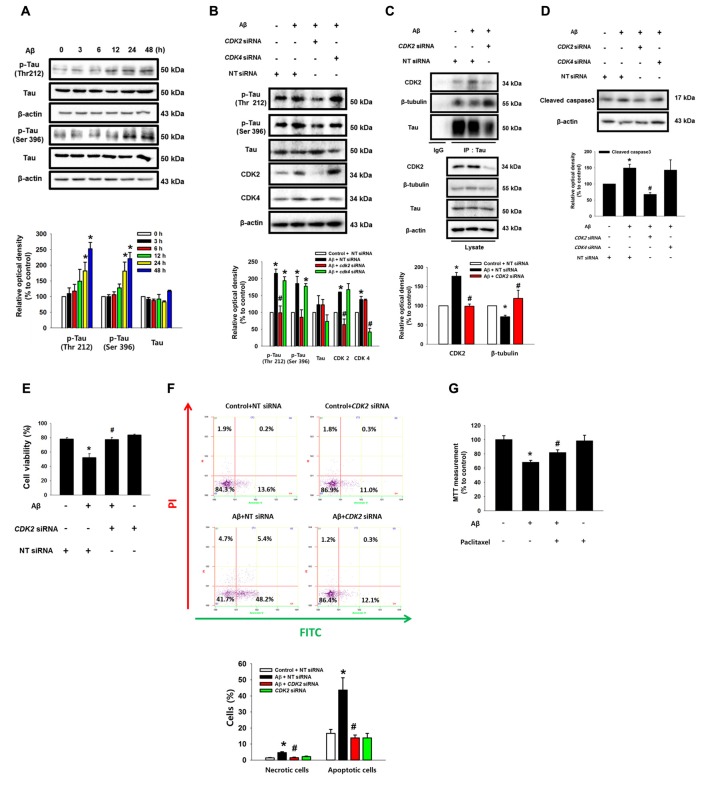
CDK2 plays a critical role in tau phosphorylation and microtubule destabilization. **(A)** SK-N-MC cells were incubated with 5 μM of Aβ for 0–48 h. Phosphorylated tau (Thr 212 and Ser 396), Tau and β-actin were detected by western blot. Cells were transfected with *CDK2*, *CDK4* and NT siRNAs for 24 h prior to Aβ treatment for 24 h. *n* = 4. **(B)** Phosphorylated tau (Thr 212 and Ser 396), tau, CDK2, CDK4 and β-actin were detected by western blot. *n* = 3. **(C)** Protein samples were immunoprecipitated with tau antibody-conjugated protein A/G agarose beads. Samples were blotted with CDK2, β-tubulin, tau and β-actin. *n* = 3. **(D)** Expressions of cleaved caspase3 and β-actin were analyzed by western blot. *n* = 3. **(E)** Cell viability was measured by trypan blue exclusion assay. Data are presented as a mean ± SE. *n* = 6. **(F)** Necrotic and apoptotic cells were counted by using annexin V/PI analysis with flow cytometry. Data are presented as a mean ± SE. *n* = 4. **(G)** Cells were pretreated with Paclitaxel (100 nM) for 30 min prior to Aβ treatment for 24 h. Cytotoxicity was measured by MTT assay at an absorbance of 545 nm using a microplate reader. Data are presented as a mean ± SE. *n* = 6. Each blot result shown is representative image. Quantitative blot data are presented as a mean ± SE. *n* = 3. *p < 0.05 vs. control, ^#^*p* < 0.05 vs. Aβ treatment.

Aβ significantly induced the phosphorylation of tau at Thr 212 and Ser 396 (Figure 6A). Interestingly, the Aβ-induced phosphorylations of tau (Thr 212, Ser 396 and Ser 262) were significantly blocked by the knockdown of CDK2 in SK-N-MC and mouse hippocampal neuron (Figure 6B and Supplementary Figures S6A,B), suggesting that tau phosphorylation induced by Aβ is CDK2-dependent. Furthermore, pretreatment with the CDK inhibitor flavopiridol also prevented the tau phosphorylation induced by Aβ in SK-N-MC cells (Supplementary Figure S7A) and primary neurons (Supplementary Figure S7B). It was noted that tau is co-immunoprecipitated with CDK2, and importantly, that these interactions were enhanced by the Aβ treatment (Figure 6C). Aβ also decreased tubulin interaction with tau, which was blocked by the knockdown of CDK2 (Figure 6C). Consistent with this, pretreatment with the CDK inhibitor flavopiridol also prevented microtubule destabilization as induced by Aβ (Supplementary Figure S7C). Finally, the silencing of CDK2 attenuated the level of cleavage of caspase-3 (Figure 6D), cytotoxicity (Figure 6E) and apoptotic cell death (Figure 6F) as stimulated by an Aβ treatment. In addition, we screened the effect of scrambled Aβ (1-42) peptide to check the nonspecific effect of Aβ. Our results showed that scrambled Aβ treatment did not affect the phosphorylations of Akt (Thr 308 and Ser 473), mTOR (Ser 2448), and tau (Thr 212 and Ser 396) and expressions of HIF1α and CDK2 (Supplementary Figure S8). These findings suggest that CDK2 plays a critical role in tau activation and in the detachment of tau from tubulins in promoting neuronal apoptosis. Interestingly, treatment with a microtubule stabilizer, paclitaxel, significantly blocked Aβ-induced neuronal cell death, although paclitaxel reduced cell viability by 20% (Figure 6G). As tau proteins aggregate after detachment from microtubules, we investigated whether autophagy is associated in any way with aggregated tau. Interestingly, the induction of autophagy with trehalose significantly inhibited the tau phosphorylation induced by Aβ but not the total level of the tau protein, suggesting that autophagy negatively regulates the accumulation of cell cycle regulatory proteins, but not tau itself (Supplementary Figure S9A). Moreover, tau proteins aggregated by Aβ did not co-localize with LC3 puncta despite the fact that autophagy was induced by trehalose (Supplementary Figure S9B). These results suggest that the accumulation of cell cycle regulatory proteins induced by Aβ is necessary for tauopathy, which leads to neuronal apoptosis.

## Discussion

**Figure 7:**
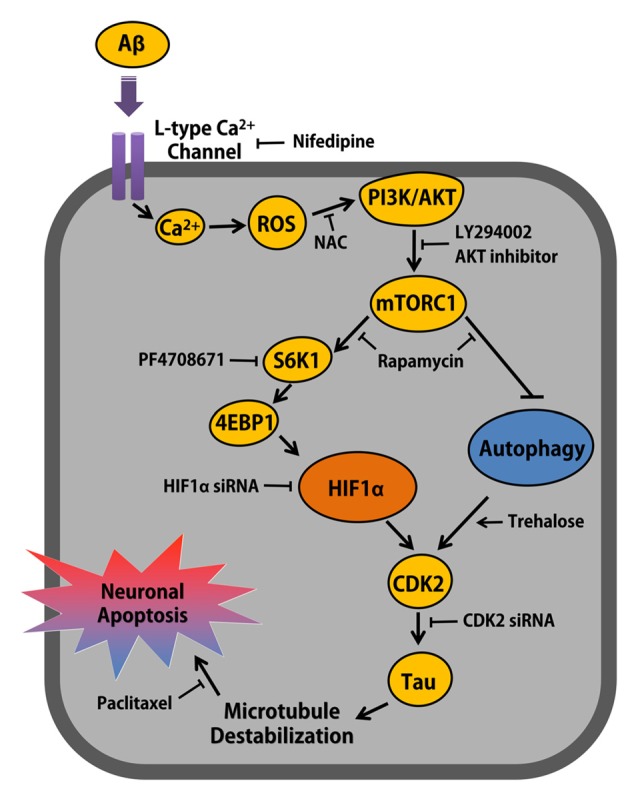
The schematic model for mechanism involved in Aβ-induced neuronal apoptosis. Aβ induces ROS production via L-type Ca^2+^ channel-mediated Ca^2+^ influx, which activates PI3K/Akt signaling pathway. mTORC1 activated by Akt up-regulates CDK2 via S6K1/4EBP1-mediated HIF1α expression and mTORC1-dependent autophagy inhibition, which is closely critical for Aβ-induced Tau phosphorylation and neuronal apoptosis in neuronal cell.

Our results here clearly show that Aβ has the ability to facilitate HIF1α synthesis and autophagy inhibition via the PI3K/Akt-dependent distinctive mTORC1 pathway, which is critical for the expression of cyclins and CDKs, where CDK2 plays a unique role in the neuronal cell death mediated by tau phosphorylation (Figure 7). Concerning the cellular mechanisms of Aβ with regard to neuronal apoptosis, our data revealed that a unique relationship between the Aβ signaling pathway and ROS production via the L-type calcium channel in the regulation of neuronal cell death. Evidence has demonstrated that oxidative stress is closely connected with several major pathological processes in AD, including tauopathy, mitochondria dysfunction and Aβ-induced neurotoxicity (Zhao and Zhao, 2013). Moreover, an increased cytosolic concentration of Ca^2+^ was shown to trigger mitochondrial damage, possibly increasing mitochondrial ROS production in various ways, such as by stimulating the TCA cycle and oxidative-phosphorylation or by blocking the respiratory chain at complex III (Brookes et al., 2004; Irigoin et al., 2009). Indeed, the L-type calcium channel was shown to possess a clear link to Ca^2+^ dysregulation induced by Aβ (Smith et al., 2005; Small et al., 2009). Thus, these results suggest that Aβ-induced ROS involves an alteration in extracellular Ca^2+^ influx, where the L-type calcium channel is an essential mediator of the Aβ signaling pathway. One of the responsive molecules associated with ROS is the mTOR, which is hyper-activated in both mild and severe AD patients (Hudson et al., 2002). While the mTOR inhibitor has been well studied for its action to ameliorate symptoms in AD models (Hudson et al., 2002), the detailed mechanisms of mTOR in promoting neuronal apoptosis and its role in AD pathogenesis remain a topic of much debate. In addition, it has been reported that PI3K/Akt/mTOR signaling pathway has differential role in mental illnesses including depression. Previous researchers reported that the PI3K/Akt/mTOR signaling regulates neuronal inflammation and apoptosis via GSK3 inhibition, which is closely associated with neuroprotection (Kitagishi et al., 2012). In the present study, we showed that ROS production generated by Aβ distinctively triggers mTORC1 activation through the PI3K/Akt signaling pathway. Given that PI3K/Akt signaling is regulated by growth factors and related receptors, we suggest that the PI3K/Akt pair has ability to activate downstream nutrient-sensing protein kinases, including mTORC1 in Aβ-treated SK-N-MC cells. In addition, it was clearly shown that ROS production induced by an Aβ treatment stimulates the PI3K/Akt-mediated mTORC1 signaling pathway in neuronal cells (Maiese et al., 2012), suggesting that the ROS-dependent activation of the PI3K/Akt/ mTORC1 pathway has a key function in Aβ-induced neuronal cell death.

A key finding of our study is that mTORC1 activated by Aβ stimulates HIF1α synthesis in promoting neuronal cell death. This result differs from that in a previous report which revealed that Aβ actually reduces the level of HIF1α via a ROS-independent mechanism in astrocytes (Schubert et al., 2009). Thus, some scientists have proposed that hypoxic preconditioning could prevent the deterioration of neuronal cells in AD as an alternative therapy (Crapper McLachlan et al., 1991; Ogunshola and Antoniou, 2009). However, many contradictory results have also been reported, showing that the manipulation of hypoxic pathways has many different outcomes (Ogunshola and Antoniou, 2009). For instance, we have shown that HIF1α binding to the *BACE1* promoter induces BACE1 expression and results in increased Aβ production in neuroblastoma cells (Lee et al., 2016). Moreover, hypoxia-mediated HIF1α signaling is involved in the amyloidogenic processing of the amyloid precursor protein, and subsequent downstream events influence the activation of the pro-death gene BNIP3, thus leading to an increased incidence of AD and neurodegeneration after cerebral ischemia and stroke (Zhang et al., 2007; Ogunshola and Antoniou, 2009). Although the reasons for the discrepancy with regard to the functional role of Aβ in the regulation of HIF1α remain unknown, one possibility in the present study is that Aβ induces the co-operative regulation of mTORC1 downstream substrates, eIF4E, 4E-BP1, and p70S6K1, to stimulate mRNA translation during the synthesis of relevant proteins, including HIF1α. This indicates that p70S6K1 activated by Aβ phosphorylates the ribosomal S6 protein and 4E-BP1, which in turn prevents the interaction between 4E-BP1 and eIF-4E ultimately to enhance the cap-dependent mRNA translation of HIF1α and HIF1α synthesis. In agreement with our current findings, mTORC1 was shown to act as a central mediator of the HIF1α translation process in promoting the expression of VEGF-A (Dodd et al., 2015). A question remains as to how Aβ induces neuronal cell death via HIF1α synthesis. We have shown that the HIF1α synthesis induced by Aβ promotes the expression of cell cycle regulatory proteins. Indeed, mTORC1 signaling acting on HIF1α was shown to promote the entering of the cell cycle, which can be harmful to neurons and which can result in neuronal apoptosis rather than replication (Bowser and Smith, 2002; Folch et al., 2012). This result is consistent with the notion that neurons are constantly struggling to keep their cell cycle in check, and negligence in this surveillance often leads to cell cycle re-entry and then to neuronal apoptosis (Herrup and Yang, 2007). Thus, our results in the present study suggest that the increased level of HIF1α synthesis is able to enhance AD pathogenesis by inducing the expression of cell cycle regulatory proteins and allow us to speculate that HIF1α inhibition provides an alternative approach for therapeutic targeting. On the other hand, we found that mTORC1 activated by Aβ facilitates autophagy inhibition to stimulate the expression of cell cycle regulatory proteins during neuronal cell death. This result is consistent with the notion that a treatment with pharmacological inhibitors of lysosomal degradation, such as bafilomycin and chloroquine, increases the expression levels of many proteins, including cell cycle regulatory proteins and even HIF1α (Hubbi et al., 2013). Indeed, it was proven that mTORC1 plays an inhibitory role in autophagy by the blocking of ULK-1 activation, which is required for autophagosome formation (Kim et al., 2011; Russell et al., 2013). Thus, these data indicate that Aβ may cause autophagy inhibition and in turn attenuate the lysosomal degradation of downstream signaling molecules in the Aβ signaling pathway. Collectively, these results provide important evidence of a pathogenic mechanism of the Aβ-meditated mTORC1 signaling pathway by which the level of cell cycle regulatory proteins is enhanced in a multifaceted manner through not only the stimulation of HIF1α synthesis but also the inhibition of autophagy.

Among cell cycle protein kinases, we identified here that Aβ facilitates CDK2 interaction with a tau protein and that CDK2 is highly involved in the processes of tau phosphorylation and microtubule destabilization during neuronal cell death. In support of our data, the tau protein was shown to have several motifs which can interact with cell cycle protein kinases, including CDK2 (Schmetsdorf et al., 2009). NMR spectroscopy analysis results also revealed that the CDK2 protein complex has the ability to phosphorylate the tau protein (Welburn and Endicott, 2005). Thus, these results present the possibility that CDK2 interacting with tau protein may be an important signaling event for tau phosphorylation induced by Aβ. Previous studies have discovered many candidate molecules that phosphorylate tau, including the mTOR, glycogen synthase kinase 3β (GSK3β) and CDK5 (Rankin et al., 2007; Caccamo et al., 2013; Kimura et al., 2014). Although we have not studied the role of Aβ on GSK3β, we found that Aβ did not have an effect on the expression of CDK5. Indeed, CDK5 was reported to play a positive role in neuronal survival under hypoxic conditions (O’Hare et al., 2005). In contrast, previous reports provided compelling evidence that CDK2 plays a critical role in the neuronal apoptosis induced by Aβ, while a specific inhibitor of CDK1 and CDK2, flavopiridol, shows neuroprotective effects (Copani et al., 1999, 2001; Folch et al., 2012). Thus, these results indicate that Aβ facilitates the interaction between CDK2 and tau protein and uniquely regulates tau phosphorylation during neuronal apoptosis as induced by Aβ. On the other hand, it is well known that tau is a neuronal-specific protein associated with microtubules and that it plays a pivotal role in the maintenance of the neuronal cytoskeleton and neurofilaments. However, given that microtubule destabilization in neuronal axons attenuates synaptic plasticity and may also lead to neuronal apoptosis and thus causing clinical symptoms of AD (Bendiske et al., 2002; Conde and Cáceres, 2009; Tu et al., 2014), our findings here suggest that Aβ induces the CDK2-mediated phosphorylation of tau and is then responsible for microtubule destabilization ultimately to promote neuronal apoptosis. Several reports have suggested that tau aggregates induced by Aβ can be removed from cells by autophagy to then be rescued from apoptosis (Bandyopadhyay et al., 2007; Flach et al., 2012; Krüger et al., 2012). However, our results in the present study revealed that autophagy induction does not affect the degradation of tau aggregates but blocks the phosphorylation of tau. These findings suggest that Aβ contributes to tauopathy via the CDK2-mediated phosphorylation of tau by preventing autophagy induction. Our results and schematic model were based on the data analyzed by quantitative western blot. Western blot-based analysis is highly depending on the specificity of antibodies. Therefore, further analysis of protein concentration and phosphorylation (such as proteomics and transcriptomics) will support our observation.

Overall, the results of this study suggest that Aβ is responsible for the AD pathogenesis by stimulating neuronal apoptosis, through which Aβ stimulates the ROS-mediated PI3K/Akt pathway to activate mTORC1, which is essential for the processes of autophagy inhibition and HIF1α synthesis. The Aβ signaling pathway through mTORC1 activation ultimately triggers the CDK2-mediated phosphorylation of tau and the destabilization of microtubules for neuronal cell death. Thus, highlighting the signaling pathways involved in the Aβ-stimulated cell death pathway may provide potential therapeutic targets for strategic modulations of AD.

## Author Contributions

KHL: conception and design, collection of data, data analysis and interpretation, manuscript writing; S-JL and HJH: conception and design, data analysis and interpretation, manuscript writing; HJL: conception and design, collection of data, data analysis and interpretation; GEC, YHJ and DIK: conception and design, data analysis and interpretation; AAG: data analysis and interpretation; JMR: conception and design.

## Conflict of Interest Statement

The authors declare that the research was conducted in the absence of any commercial or financial relationships that could be construed as a potential conflict of interest.

## Footnotes

^1^https://imagej.nih.gov/ij/download.html

## References

[B1] Alonso A. D. Di Clerico J. Li B. Corbo C. P. Alaniz M. E. Grundke-Iqbal I.. (2010). Phosphorylation of tau at Thr212, Thr231, and Ser262 combined causes neurodegeneration. J. Biol. Chem. 285, 30851–30860. 10.1074/jbc.M110.110957 20663882 PMC2945578

[B2] Arendt T. Brückner M. K. (2007). Linking cell-cycle dysfunction in Alzheimer’s disease to a failure of synaptic plasticity. Biochim. Biophys. Acta 1772, 413–421. 10.1016/j.bbadis.2006.12.005 17236750

[B3] Bandyopadhyay B. Li G. Yin H. Kuret J. (2007). Tau aggregation and toxicity in a cell culture model of tauopathy. J. Biol. Chem. 282, 16454–16464. 10.1074/jbc.M700192200 17428800

[B4] Bendiske J. Caba E. Brown Q. B. Bahr B. A. (2002). Intracellular deposition, microtubule destabilization, and transport failure: an “early” pathogenic cascade leading to synaptic decline. J. Neuropathol. Exp. Neurol. 61, 640–650. 10.1093/jnen/61.7.640 12125743

[B5] Bowser R. Smith M. A. (2002). Cell cycle proteins in Alzheimer’s disease: plenty of wheels but no cycle. J. Alzheimers Dis. 4, 249–254. 10.3233/JAD-2002-4316 12226545

[B6] Brookes P. S. Yoon Y. Robotham J. L. Anders M. W. Sheu S. S. (2004). Calcium, ATP, and ROS: a mitochondrial love-hate triangle. Am. J. Physiol. Cell Physiol. 287, C817–C833. 10.1152/ajpcell.00139.2004 15355853

[B7] Caccamo A. Magrì A. Medina D. X. Wisely E. V. López-Aranda M. F. Silva A. J.. (2013). mTOR regulates tau phosphorylation and degradation: implications for Alzheimer’s disease and other tauopathies. Aging Cell 12, 370–380. 10.1111/acel.12057 23425014 PMC3655115

[B8] Chan A. S. Ng L. W. Poon L. S. Chan W. W. Wong Y. H. (2007). Dopaminergic and adrenergic toxicities on SK-N-MC human neuroblastoma cells are mediated through G protein signaling and oxidative stress. Apoptosis 12, 167–179. 10.1007/s10495-006-0524-8 17136323

[B9] Conde C. Cáceres A. (2009). Microtubule assembly, organization and dynamics in axons and dendrites. Nat. Rev. Neurosci. 10, 319–332. 10.1038/nrn2631 19377501

[B10] Copani A. Caraci F. Hoozemans J. J. Calafiore M. Sortino M. A. Nicoletti F. (2007). The nature of the cell cycle in neurons: focus on a “non-canonical” pathway of DNA replication causally related to death. Biochim. Biophys. Acta 1772, 409–412. 10.1016/j.bbadis.2006.10.016 17196375

[B11] Copani A. Condorelli F. Caruso A. Vancheri C. Sala A. Giuffrida Stella A. M.. (1999). Mitotic signaling by β-amyloid causes neuronal death. FASEB J. 13, 2225–2234. 10593870

[B12] Copani A. Uberti D. Sortino M. A. Bruno V. Nicoletti F. Memo M. (2001). Activation of cell-cycle-associated proteins in neuronal death: a mandatory or dispensable path? Trends Neurosci. 24, 25–31. 10.1016/s0166-2236(00)01663-5 11163884

[B13] Crapper McLachlan D. R. Dalton A. J. Kruck T. P. Bell M. Y. Smith W. L. Kalow W.. (1991). Intramuscular desferrioxamine in patients with Alzheimer’s disease. Lancet 337, 1304–1308. 10.1016/0140-6736(91)92978-b 1674295

[B14] Dodd K. M. Yang J. Shen M. H. Sampson J. R. Tee A. R. (2015). mTORC1 drives HIF-1α and VEGF-A signalling via multiple mechanisms involving 4E-BP1, S6K1 and STAT3. Oncogene 34, 2239–2250. 10.1038/onc.2014.164 24931163 PMC4172452

[B15] Flach K. Hilbrich I. Schiffmann A. Gärtner U. Kruger M. Leonhardt M.. (2012). Tau oligomers impair artificial membrane integrity and cellular viability. J. Biol. Chem. 287, 43223–43233. 10.1074/jbc.M112.396176 23129775 PMC3527910

[B16] Folch J. Junyent F. Verdaguer E. Auladell C. Pizarro J. G. Beas-Zarate C.. (2012). Role of cell cycle re-entry in neurons: a common apoptotic mechanism of neuronal cell death. Neurotox. Res. 22, 195–207. 10.1007/s12640-011-9277-4 21965004

[B17] Frade J. M. Ovejero-Benito M. C. (2015). Neuronal cell cycle: the neuron itself and its circumstances. Cell Cycle 14, 712–720. 10.1080/15384101.2015.1004937 25590687 PMC4418291

[B18] He C. Klionsky D. J. (2009). Regulation mechanisms and signaling pathways of autophagy. Annu. Rev. Genet. 43, 67–93. 10.1146/annurev-genet-102808-114910 19653858 PMC2831538

[B19] Herrup K. Yang Y. (2007). Cell cycle regulation in the postmitotic neuron: oxymoron or new biology? Nat. Rev. Neurosci. 8, 368–378. 10.1038/nrn2124 17453017

[B20] Hubbi M. E. Hu H. Kshitiz Ahmed I. Levchenko A. Semenza G. L. (2013). Chaperone-mediated autophagy targets hypoxia-inducible factor-1α (HIF-1α) for lysosomal degradation. J. Biol. Chem. 288, 10703–10714. 10.1074/jbc.M112.414771 23457305 PMC3624450

[B21] Hudson C. C. Liu M. Chiang G. G. Otterness D. M. Loomis D. C. Kaper F.. (2002). Regulation of hypoxia-inducible factor 1α expression and function by the mammalian target of rapamycin. Mol. Cell. Biol. 22, 7004–7014. 10.1128/mcb.22.20.7004-7014.2002 12242281 PMC139825

[B22] Hyman B. T. Augustinack J. C. Ingelsson M. (2005). Transcriptional and conformational changes of the tau molecule in Alzheimer’s disease. Biochim. Biophys. Acta 1739, 150–157. 10.1016/j.bbadis.2004.06.015 15615634

[B23] Irigoin F. Inada N. M. Fernandes M. P. Piacenza L. Gadelha F. R. Vercesi A. E.. (2009). Mitochondrial calcium overload triggers complement-dependent superoxide-mediated programmed cell death in Trypanosoma cruzi. Biochem. J. 418, 595–604. 10.1042/BJ20081981 19053945

[B24] Johnson G. V. Stoothoff W. H. (2004). Tau phosphorylation in neuronal cell function and dysfunction. J. Cell Sci. 117, 5721–5729. 10.1242/jcs.01558 15537830

[B26] Kim J. Kundu M. Viollet B. Guan K. L. (2011). AMPK and mTOR regulate autophagy through direct phosphorylation of Ulk1. Nat. Cell Biol. 13, 132–141. 10.1038/ncb2152 21258367 PMC3987946

[B25] Kim D. I. Lee K. H. Oh J. Y. Kim J. S. Han H. J. (2017). Relationship between β-amyloid and mitochondrial dynamics. Cell. Mol. Neurobiol. 37, 955–968. 10.1007/s10571-016-0434-4 27766447 PMC11482120

[B27] Kimura T. Ishiguro K. Hisanaga S. (2014). Physiological and pathological phosphorylation of tau by Cdk5. Front. Mol. Neurosci. 7:65. 10.3389/fnmol.2014.00065 25076872 PMC4097945

[B28] Kitagishi Y. Kobayashi M. Kikuta K. Matsuda S. (2012). Roles of PI3K/AKT/GSK3/mTOR pathway in cell signaling of mental illnesses. Depress. Res. Treat. 2012:752563. 10.1155/2012/752563 23320155 PMC3535741

[B29] Klionsky D. J. Emr S. D. (2000). Autophagy as a regulated pathway of cellular degradation. Science 290, 1717–1721. 10.1126/science.290.5497.1717 11099404 PMC2732363

[B30] Krüger U. Wang Y. Kumar S. Mandelkow E.-M. (2012). Autophagic degradation of tau in primary neurons and its enhancement by trehalose. Neurobiol. Aging 33, 2291–2305. 10.1016/j.neurobiolaging.2011.11.009 22169203

[B31] Kuo Y.-C. Chou P.-R. (2014). Neuroprotection against degeneration of SK-N-MC cells using neuron growth factor-encapsulated liposomes with surface cereport and transferrin. J. Pharm. Sci. 103, 2484–2497. 10.1002/jps.24081 25041794

[B32] Lee H. J. Ryu J. M. Jung Y. H. Lee S. J. Kim J. Y. Lee S. H.. (2016). High glucose upregulates BACE1-mediated Aβ production through ROS-dependent HIF-1α and LXRα/ABCA1-regulated lipid raft reorganization in SK-N-MC cells. Sci. Rep. 6:36746. 10.1038/srep36746 27829662 PMC5103190

[B33] Liang J. H. Jia J. P. (2014). Dysfunctional autophagy in Alzheimer’s disease: pathogenic roles and therapeutic implications. Neurosci. Bull. 30, 308–316. 10.1007/s12264-013-1418-8 24610177 PMC5562662

[B34] Liu S. L. Wang C. Jiang T. Tan L. Xing A. Yu J. T. (2016). The role of Cdk5 in Alzheimer’s disease. Mol. Neurobiol. 53, 4328–4342. 10.1007/s12035-015-9369-x 26227906

[B35] Maiese K. Chong Z. Z. Wang S. Shang Y. C. (2012). Oxidant stress and signal transduction in the nervous system with the PI 3-K, Akt, and mTOR cascade. Int. J. Mol. Sci. 13, 13830–13866. 10.3390/ijms131113830 23203037 PMC3509553

[B36] Moh C. Kubiak J. Z. Bajic V. P. Zhu X. Smith M. A. Lee H. G. (2011). Cell cycle deregulation in the neurons of Alzheimer’s disease. Results Probl. Cell Differ. 53, 565–576. 10.1007/978-3-642-19065-0_23 21630160 PMC5925746

[B37] Ogunshola O. O. Antoniou X. (2009). Contribution of hypoxia to Alzheimer’s disease: is HIF-1α a mediator of neurodegeneration? Cell. Mol. Life Sci. 66, 3555–3563. 10.1007/s00018-009-0141-0 19763399 PMC11115623

[B38] O’Hare M. J. Kushwaha N. Zhang Y. Aleyasin H. Callaghan S. M. Slack R. S.. (2005). Differential roles of nuclear and cytoplasmic cyclin-dependent kinase 5 in apoptotic and excitotoxic neuronal death. J. Neurosci. 25, 8954–8966. 10.1523/jneurosci.2899-05.2005 16192386 PMC6725602

[B39] Rankin C. A. Sun Q. Gamblin T. C. (2007). Tau phosphorylation by GSK-3β promotes tangle-like filament morphology. Mol. Neurodegener. 2:12. 10.1186/1750-1326-2-12 17598919 PMC1936422

[B40] Russell R. C. Tian Y. Yuan H. Park H. W. Chang Y. Y. Kim J.. (2013). ULK1 induces autophagy by phosphorylating Beclin-1 and activating VPS34 lipid kinase. Nat. Cell Biol. 15, 741–750. 10.1038/ncb2757 23685627 PMC3885611

[B41] Schmetsdorf S. Arnold E. Holzer M. Arendt T. Gärtner U. (2009). A putative role for cell cycle-related proteins in microtubule-based neuroplasticity. Eur. J. Neurosci. 29, 1096–1107. 10.1111/j.1460-9568.2009.06661.x 19302146

[B42] Schubert D. Soucek T. Blouw B. (2009). The induction of HIF-1 reduces astrocyte activation by amyloid β peptide. Eur. J. Neurosci. 29, 1323–1334. 10.1111/j.1460-9568.2009.06712.x 19519624 PMC2752839

[B43] Seibenhener M. L. Wooten M. W. (2012). Isolation and culture of hippocampal neurons from prenatal mice. J. Vis. Exp. 65:3634. 10.3791/3634 22871921 PMC3476399

[B44] Shaykhalishahi H. Taghizadeh M. Yazdanparast R. Chang Y. T. (2010). Anti-amyloidogenic effect of AA3E2 attenuates β -amyloid induced toxicity in SK-N-MC cells. Chem. Biol. Interact. 186, 16–23. 10.1016/j.cbi.2010.03.042 20359466

[B45] Small D. H. Gasperini R. Vincent A. J. Hung A. C. Foa L. (2009). The role of A β -induced calcium dysregulation in the pathogenesis of Alzheimer’s disease. J. Alzheimers Dis. 16, 225–233. 10.3233/JAD-2009-0951 19221414

[B46] Smith I. F. Green K. N. LaFerla F. M. (2005). Calcium dysregulation in Alzheimer’s disease: recent advances gained from genetically modified animals. Cell Calcium 38, 427–437. 10.1016/j.ceca.2005.06.021 16125228

[B47] Son J. H. Shim J. H. Kim K. H. Ha J. Y. Han J. Y. (2012). Neuronal autophagy and neurodegenerative diseases. Exp. Mol. Med. 44, 89–98. 10.3858/emm.2012.44.2.031 22257884 PMC3296817

[B48] Spires-Jones T. L. Hyman B. T. (2014). The intersection of amyloid β and tau at synapses in Alzheimer’s disease. Neuron 82, 756–771. 10.1016/j.neuron.2014.05.004 24853936 PMC4135182

[B49] Sun Y. X. Ji X. Mao X. Xie L. Jia J. Galvan V.. (2014). Differential activation of mTOR complex 1 signaling in human brain with mild to severe Alzheimer’s disease. J. Alzheimers Dis. 38, 437–444. 10.3233/JAD-131124 23979023

[B50] Tramutola A. Triplett J. C. Di Domenico F. Niedowicz D. M. Murphy M. P. Coccia R.. (2015). Alteration of mTOR signaling occurs early in the progression of Alzheimer disease (AD): analysis of brain from subjects with pre-clinical AD, amnestic mild cognitive impairment and late-stage AD. J. Neurochem. 133, 739–749. 10.1111/jnc.13037 25645581

[B51] Tu S. Okamoto S. Lipton S. A. Xu H. (2014). Oligomeric Aβ-induced synaptic dysfunction in Alzheimer’s disease. Mol. Neurodegener. 9:48. 10.1186/1750-1326-9-483 25394486 PMC4237769

[B52] Wang C. Yu J. T. Miao D. Wu Z. C. Tan M. S. Tan L. (2014). Targeting the mTOR signaling network for Alzheimer’s disease therapy. Mol. Neurobiol. 49, 120–135. 10.1007/s12035-013-8505-8 23853042

[B53] Welburn J. Endicott J. (2005). Methods for preparation of proteins and protein complexes that regulate the eukaryotic cell cycle for structural studies. Methods Mol. Biol. 296, 219–235. 10.1385/1-59259-857-9:219 15576935

[B54] Wischik C. M. Edwards P. C. Lai R. Y. Gertz H. N. Xuereb J. H. Paykel E. S.. (1995). Quantitative analysis of tau protein in paired helical filament preparations: implications for the role of tau protein phosphorylation in PHF assembly in Alzheimer’s disease. Neurobiol. Aging 16, 409–417. 10.1016/0197-4580(95)97327-d 7566350

[B55] Zhang S. Zhang Z. Sandhu G. Ma X. Yang X. Geiger J. D.. (2007). Evidence of oxidative stress-induced BNIP3 expression in amyloid β neurotoxicity. Brain Res. 1138, 221–230. 10.1016/j.brainres.2006.12.086 17274962

[B56] Zhao Y. Zhao B. (2013). Oxidative stress and the pathogenesis of Alzheimer’s disease. Oxid. Med. Cell. Longev. 2013:316523. 10.1155/2013/316523 23983897 PMC3745981

