# Supplementary material for: Amyloid β1-42 (Aβ1-42) Induces the CDK2-Mediated Phosphorylation of Tau through the Activation of the mTORC1 Signaling Pathway While Promoting Neuronal Cell Death
Source: Front Mol Neurosci. 2017 Jul 24;10:229. doi: 10.3389/fnmol.2017.00229 (PMC5522873; doi:10.3389/fnmol.2017.00229)
Supplement: Supplementary file 1 [file Data_Sheet_1.docx]

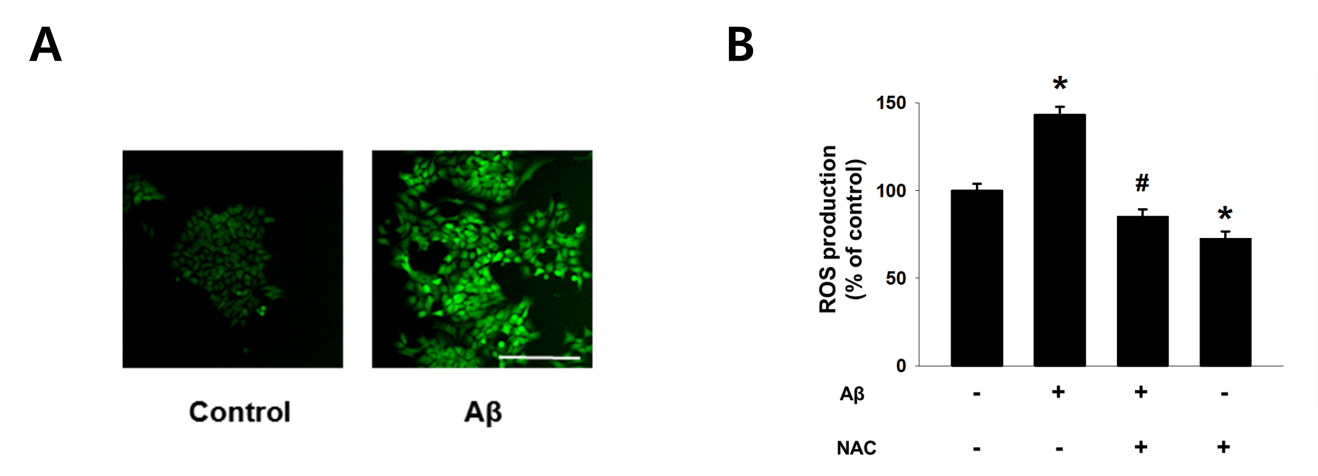


**Supplementary Figure S1. Effect of Aβ and NAC on ROS production.** (A) SK-N-MC cells were incubated with Aβ (5 μM) for 24 h. To detect ROS production, cells were incubated with H_2_DCF-DA for 1 h, and treated with Aβ. *n* = 3. Scale bars, 200 μm (magnification, × 200). (B) SK-N-MC cells were pretreated with NAC (1 mM) for 30 min prior to Aβ treatment (5 µM) for 24 h. ROS production measured by using H_2_DCF-DA staining. Data represent the mean ± SE. *n* = 6. *^*^p<0.05* versus control, *^#^p<0.05* versus Aβ treatment.


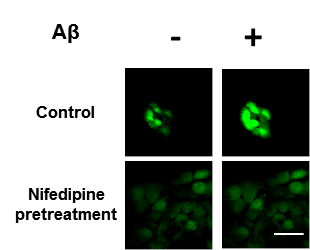


**Supplementary Figure S2. Role of L-type calcium channel in Aβ-induced calcium influx.** SK-N-MC cells were pretreated with nifedipine (10 μM) for 30 min prior to Aβ treatment. The changes in ROS production were observed using confocal microscopy. Scale bars, 100 μm (magnification, × 400). *n* = 3.


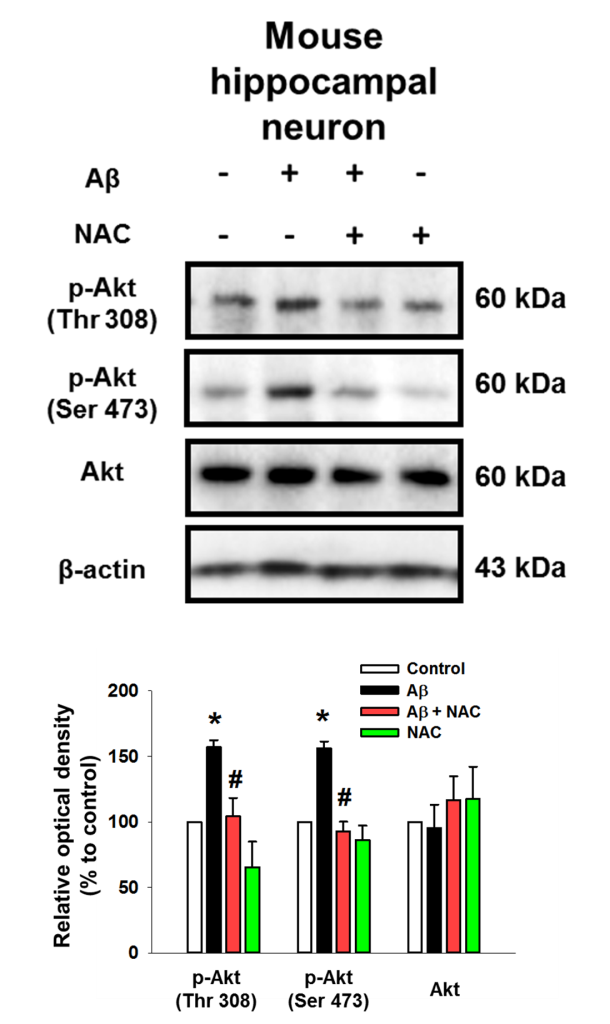


**Supplementary Figure S3. Effect of NAC on Aβ-induced Akt phosphorylation in mouse hippocampal neuron.** Cells were pretreated with NAC (1 mM) for 30 min prior to Aβ (5 uM) treatment for 24 h. p-Akt (Thr 308 and Ser 473), Akt and β-actin were detected by western blot. All blot images are representative, respectively. Quantitative data are presented as a mean ± SE. *n* = 3. *^*^p<0.05* versus control, *^#^p<0.05* versus Aβ treatment.


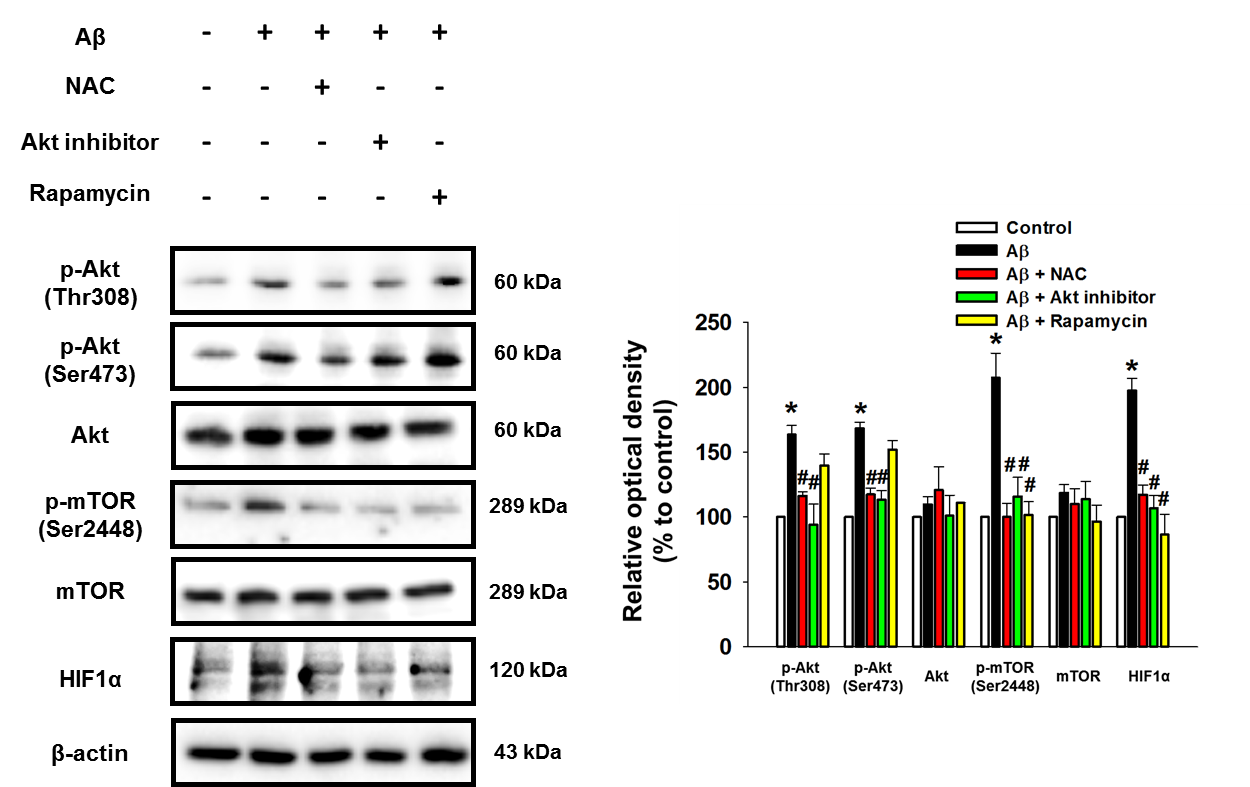


**Supplementary Figure S4. Sequential activation Akt/mTOR/HIF-1α by Aβ in SK-N-MC.** SK-N-MC cells were pretreated NAC (1 mM) or Akt inhibitor (10 μM ) or rapamcyin (10 nM) for 30 min prior to Aβ (5 μM) for 24 h. p-Akt (Thr 308 and Ser 473), Akt, p-mTOR (Ser 2448), mTOR, HIF1α and β-actin were detected by western blot. Western blot result shown is representative image. Quantitative blot data are presented as a mean ± SE. *n* = 4. All blot images are representative, respectively. *^*^p<0.05* versus control, *^#^p<0.05* versus Aβ treatment.


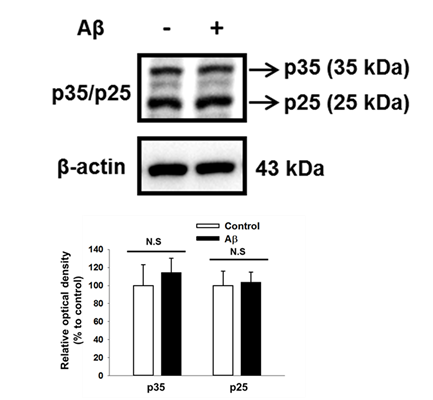


**Supplementary Figure S5. Effect of Aβ on p35 cleavage in SK-N-MC.** SK-N-MC cells were incubated with Aβ (5 μM) for 24 h. p35/p25 and β-actin were detected by western blot. Western blot result shown is representative image. Quantitative data are presented as a mean ± SE. *n* = 5. N.S indicates not statistically significant.


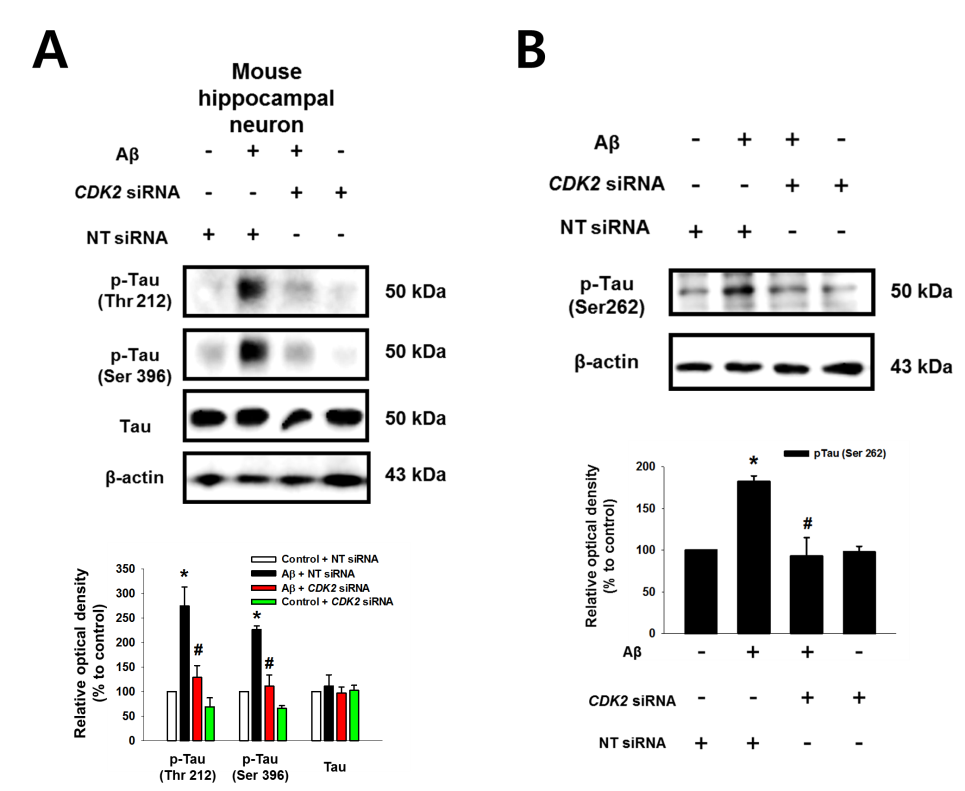


**Supplemtentary Figure S6. Role of CDK2 in Aβ-induced tau phosphorylation in mouse hippocampal neuron.** (A,B) Cells were transfected with mouse *Cdk2* siRNA or NT siRNA for 24 h prior to Aβ treatment (5 uM) for 24 h. *n* = 3. (A) p-tau (Thr 212 and Ser 396), tau and β-actin in mouse primary neuron were analyzed by western blot. (B) p-tau (Ser 262) and β-actin in SK-N-MCs were detected by western blot. *n* = 3. All blot images are representative, respectively. Quantitative data are presented as a mean ± SE. *^*^p<0.05* versus control, *^#^p<0.05* versus Aβ treatment.


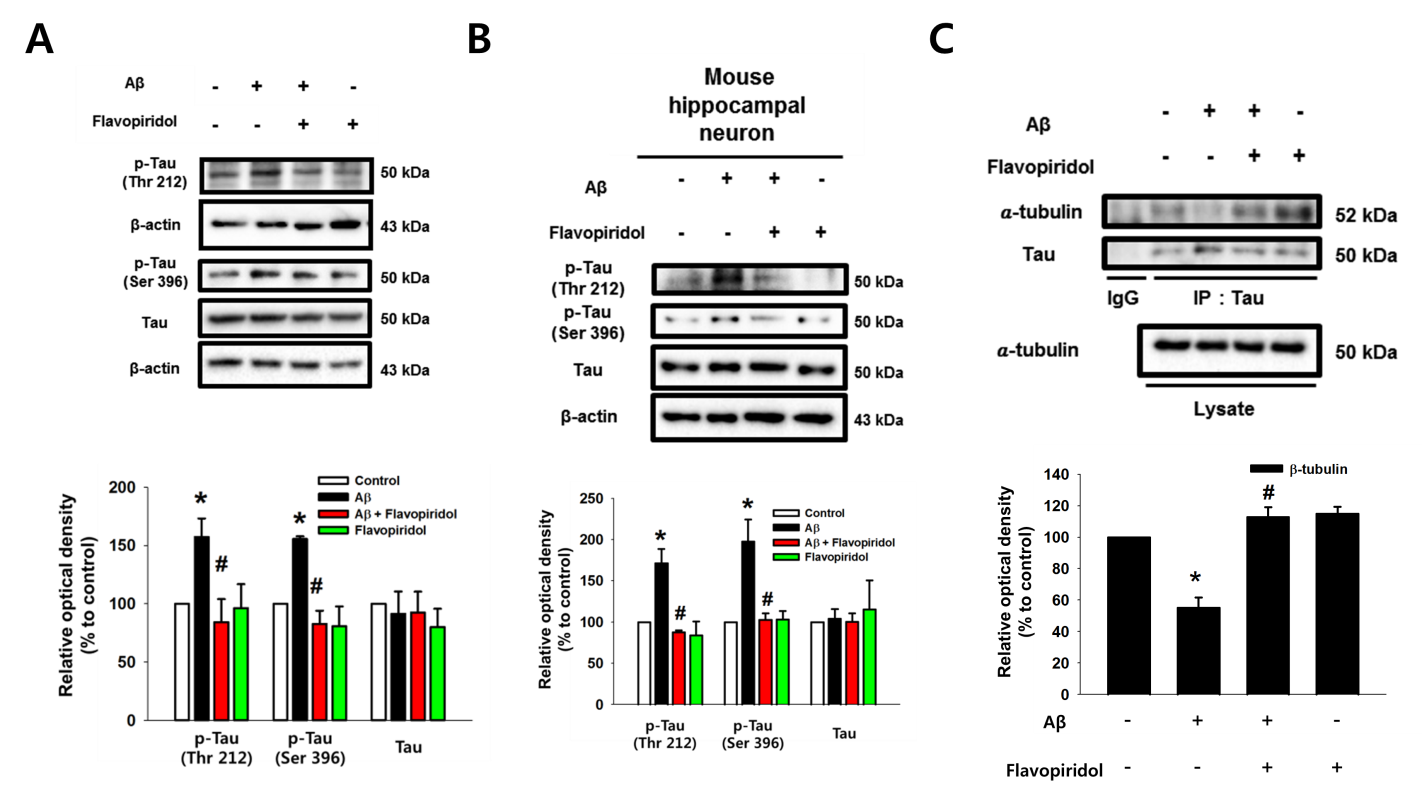


**Supplementary Figure S7. Role of CDK inhibitor Flavopiridol in Aβ-induced tau phosphorylation and microtubule destabilization.** (A,B) SK-N-MC cells and mouse hippocampal neurons were pretreated with flavopridol (1 nM) for 30 min prior to Aβ treatment for 24 h. Samples were blotted with p-tau (Thr 212 and Ser 396), tau and β-actin specific antibodies. (C) Protein samples from SK-N-MC cells were immunoprecipitated with tau specific antibody-conjugated protein A/G agarose beads. Samples were blotted with α-tubulin and tau specific antibodies. Each blot result shown is representative image. Quantitative blot data are presented as a mean ± SE. *n* = 3-6. *^*^p<0.05* versus control, *^#^p<0.05* versus Aβ treatment.


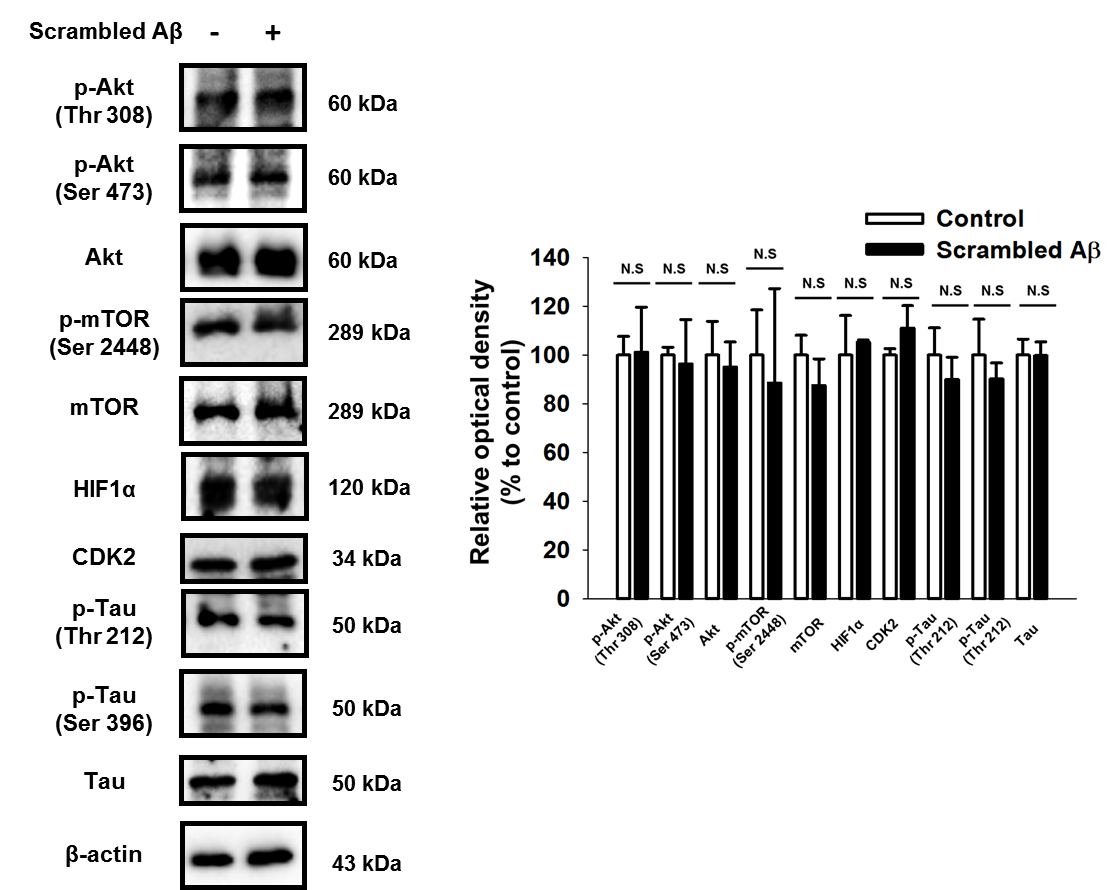


**Supplementary Figure S8. Effect of scrambled Aβ on Akt, mTOR, HIF1α, CDK2and tau.** SK-N-MC cells were treated with scrambled peptide of Aβ (5 μM) for 24 h. p-Akt (Thr 308 and Ser 473), Akt, p-mTOR (Ser 2448), mTOR, HIF1α, CDK2, p-tau (Thr 212 and Ser 396), tau and β-actin were detected by western blot. All blot images are representative. Quantitative data are presented as a mean ± SE. *n* = 5. All blot images are representative, respectively. N.S indicates not statistically significant.


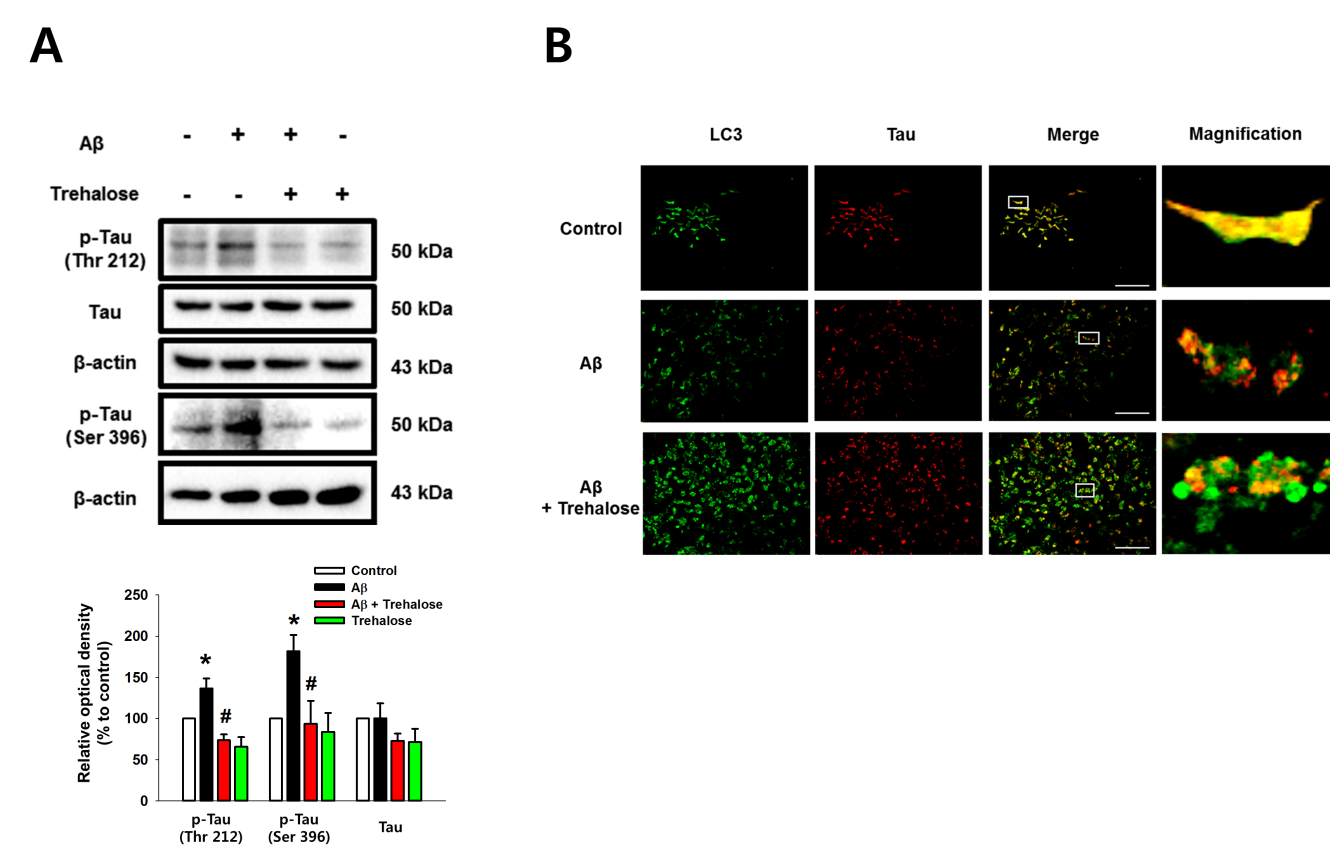


**Supplementary Figure S9. Role of autophagy in tau phosphorylation**. SK-N-MC cells were incubated with trehalose (10 μM) for 30 min prior to Aβ (5 μM) for 24 h. (A) Phosphorylated tau (Thr 212 and Ser 396), tau and β-actin were analyzed by western blot. (B) Expression of LC3 and tau in SK-N-MC cells was visualized by confocal microscopy. Scale bars, 50 μm (magnification × 600). Western blot result shown is representative image. Quantitative blot data are presented as a mean ± SE. *n* = 3. *^*^p<0.05* versus control, *^#^p<0.05* versus Aβ treatment.

| Gene | Identification | Sequence (5'-3') | Size (bp) |
| --- | --- | --- | --- |
| *CDK5* | Sense | TGAAGCCCCAGAACCTGCTA | 243 |
|  | Antisense | CATCATTGCCGGGAAAAAGA |  |
| *P35* | Sense | GCGTCATGGCTCAAGGATTC | 233 |
|  | Antisense | GTTCCCCAAAGTGGGAGGTC |  |
| *P39* | Sense | ACCCCTCTCCCCTGATCAAA | 155 |
|  | Antisense | GCATGCAACACGTTGGAAAA |  |
| *ACTB* | Sense | AACCGCGAGAAGATGACC | 351 |
|  | Antisense | AGCAGCCGTGGCCATCTC |  |

**Supplementary Table S1. Sequences of primers used for RT-PCR and real-time PCR**

**Supplementary Table S2. Sequences of siRNAs used for gene silencing**

| Target gene | Sequence 5'-3' |
| --- | --- |
| *HIF1A* | GCCGCUCAAUUUAUGAAUATT  UAUUCAUAAAUUGAGCGGCTT |
|  | GCCUCUUUGACAAACUUAATT  UUAAGUUUGUCAAAGAGGCTT |
|  | CCACCACUGAUGAAUUAAATT  UUUAAUUCAUCAGUGGUGGTT |
|  | GCUGGAGACACAAUCAUAUTT  AUAUGAUUGUGUCUCCAGCTT |
| *CDK2* | GAGCUUAACCAUCCUAAUA |
|  | GAAACAAGUUGACGGGAGA |
|  | GGAGUUACUUCUAUGCCUG |
|  | GGGCCUAGCUUUCUGCCAU |
| Mouse *Cdk2* | GAACUUAAUCACCCUAAUA |
|  | GGACGGAGCUUGUUAUCGC |
|  | GAGAAGUUGUGGCGCUUAA |
|  | ACCAGGACCUCAAGAAAUU |
| *CDK4* | CAAGGUAACCCUGGUGUUU |
|  | GAGCUCUGCAGCACUCUUA |
|  | CAGCACAGUUCGUGAGGUG |
|  | GCACUUACACCCGUGGUUG |
| Non-targeting | UAGCGACUAAACACAUCAA |
|  | UAAGGCUAUGAAGAGAUAC |
|  | AUGUAUUGGCCUGUAUUAG |
|  | AUGAACGUGAAUUGCUCAA |
